# “Hydridic Hydrogen-Bond Donors” Are Not Hydrogen-Bond Donors

**DOI:** 10.1021/jacs.4c07821

**Published:** 2024-09-03

**Authors:** Lucas de Azevedo Santos, Pascal Vermeeren, F. Matthias Bickelhaupt, Célia Fonseca Guerra

**Affiliations:** † Department of Chemistry and Pharmaceutical Sciences, AIMMS, Vrije Universiteit Amsterdam, De Boelelaan 1108, 1081 HZ Amsterdam, The Netherlands; ‡ Institute for Molecules and Materials, Radboud University, Heyendaalseweg 135, 6525 AJ Nijmegen, The Netherlands; § Department of Chemical Sciences, University of Johannesburg, Auckland Park, Johannesburg 2006, South Africa

## Abstract

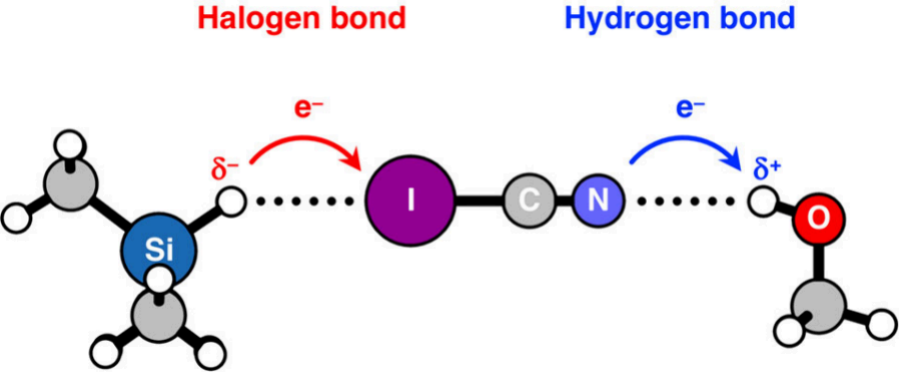

Herein, we dismiss a recent proposal by Civiš, Hobza, and co-workers to modify the IUPAC definition of hydrogen bonds in order to expand the scope from protonic Y–H^δ+^ to hydridic Y–H^δ−^ hydrogen-bond donor fragments [*J. Am. Chem. Soc.***2023**, *145*, 8550]. Based on accurate Kohn–Sham molecular orbital (KS-MO) analyses, we falsify the conclusion that interactions involving protonic and hydridic hydrogens are both hydrogen bonds; they are not. Instead, our quantitative KS-MO, energy decomposition, and Voronoi deformation density analyses reveal two fundamentally different bonding mechanisms for protonic Y–H^δ+^ and hydridic Y–H^δ−^ fragments which go with charge transfer in opposite directions. On one hand, we confirm the IUPAC definition for regular hydrogen bonds in the case of protonic Y–H^δ+^ fragments. On the other hand, complexes involving Y–H^δ−^ fragments are, in fact, acceptors in other well-known families of Lewis-acid/base interactions, such as halogen bonds, chalcogen bonds, and pnictogen bonds. These mechanisms lead to the same spectroscopic phenomenon in both the Y–H^δ+^ and Y–H^δ−^ fragments, that is, the redshift in the Y–H stretching frequency, which is, thus, not an exclusive indicator for hydrogen bonding.

## Introduction

The hydrogen bond is a key chemical interaction in biological, supramolecular, and organic chemistry.^1^ IUPAC defines the hydrogen bond (HB) as an attractive interaction between a hydrogen atom from a molecule or a molecular fragment Y–H, in which Y is *more* electronegative than H, and an atom or group of atoms (Z), in which there is evidence of bond formation (i.e., a Y–H···Z hydrogen-bonded complex).^2^ According to molecular orbital (MO) theory, the reason behind the stability of the HB is twofold: (i) the electrostatic attraction between the protonic hydrogen in the Y–H^δ+^ fragment and Z; and (ii) the covalent donor–acceptor interaction stemming from the charge transfer from the HOMO of Z into the empty σ* Y–H antibonding orbital.^3^ The covalency of HB is manifested in the characteristic Y–H bond elongation and a decrease, i.e., redshift, in the vibrational frequency associated with the Y–H bond stretching mode.^3b,4^

Hydridic hydrogens are also known to engage in intramolecular interactions that do not fit the IUPAC definition of the HB. For example, in the case of the charge inverted hydrogen bonds (CIHB),^5^ a designation coined by Jabłoński to cover systems of the type Y–H^δ−^···Z, in which Y is *less* electronegative than H and Z is an electron-deficient fragment. In this point of view, hydrogen bonding refers to all interactions involving a hydrogen atom that can either be protonic, forming a regular HB, or hydridic, forming a CIHB. Recently, Civiš, Hobza, and co-workers proposed a generalization of the IUPAC definition of HB in order to cover both the protonic and hydridic forms within the same definition.^6^ According to the authors, CIHBs still hold many important features of the HB, such as the donation of charge into the σ* Y–H^δ−^ antibonding orbital and the elongation, i.e., redshift, of the Y–H^δ−^ bond. This implies that, in CIHB, hydridic hydrogens behave as Lewis acids and the electron-deficient Z fragment behaves as a Lewis base; thus, the bonding mechanism of HB and CIHB would be the same.

**Scheme 1:**
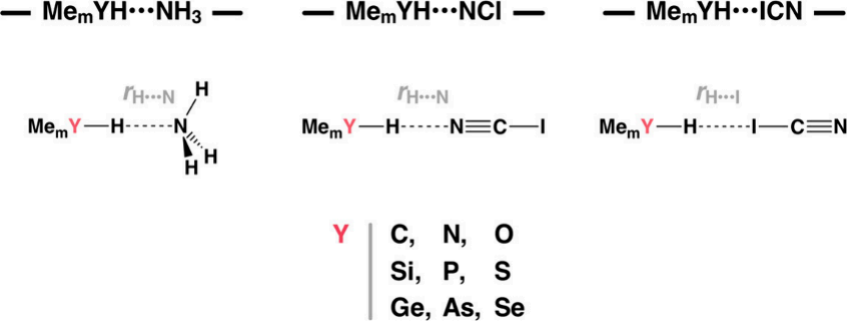
Schematic Me_m_YH···NH_3_, Me_m_YH···NCI, and Me_m_YH···NCI Model Complexes (Y = C, Si, Ge, N, P, As, O, S, Se; m = 3, 2, 1).

In this work, we challenge the idea that hydridic hydrogen bonds involve only an inversely polarized H-bond donor but otherwise are, in essence, electronically similar to protonic hydrogen bonds. To this end, we analyze the bonding mechanism of a series of Me_m_YH···NH_3_, Me_m_YH···NCI, and Me_m_YH···ICN complexes (Y = C, Si, Ge, N, P, As, O, S, Se; m = 3, 2, 1), using Kohn–Sham molecular orbital (KS-MO) theory (Scheme 1). Our model systems feature Y–H groups with protonic and hydridic hydrogens and allow for a systematic and quantitative comparison of how bonding mechanisms change in nature along the series. We show that true hydrogen bonds, i.e., in which the H-bond acts as a Lewis acid accepting charge in its σ* Y–H LUMO, can *only* be formed with protonic hydrogens. On the contrary, complexes involving hydridic hydrogens lead to a completely different bonding mechanism in which the molecular fragment containing the hydrogen atom acts as a Lewis base, not as a Lewis acid.^7^ Thus, CIHB is not a correct designation for interactions involving hydridic hydrogens, and hence, there is no need to change the IUPAC definition of the hydrogen bond.

## Results and Discussion

### The Me_m_YH Fragments

**Figure 1:**
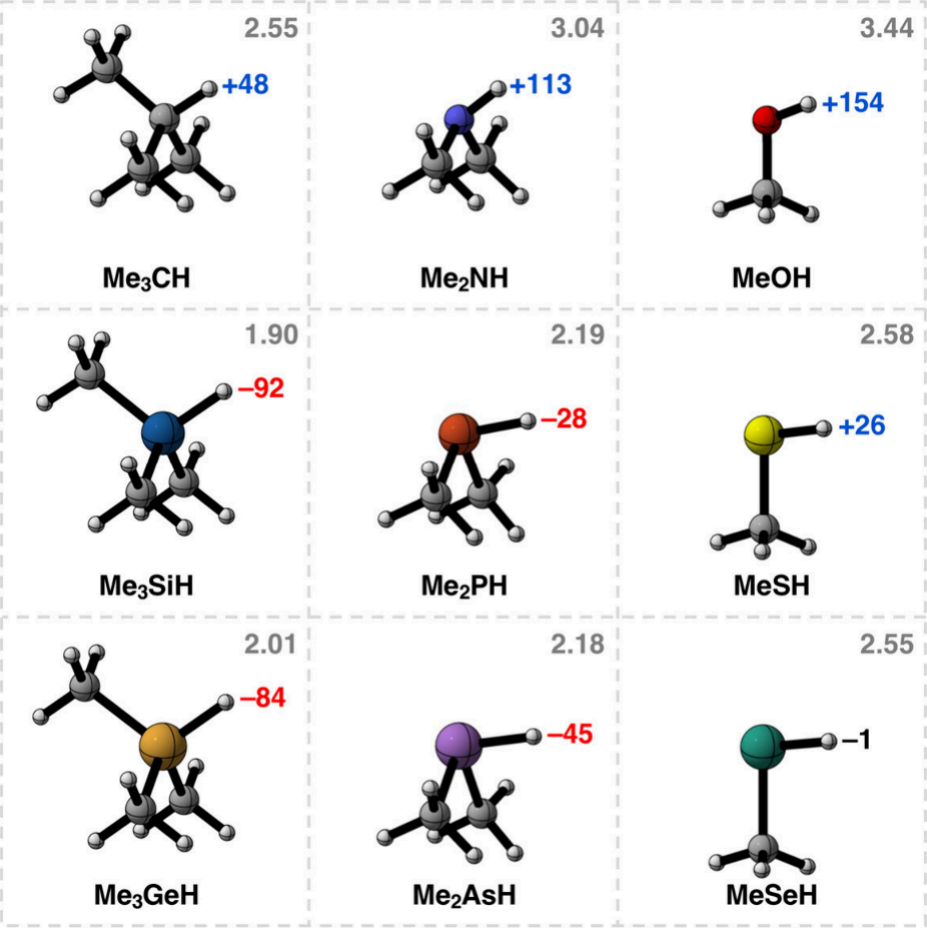
Equilibrium geometries, Voronoi deformation density charges (in milli-electrons) on the indicated hydrogen atom, and the electronegativity of Y on the Pauling scale^7^ (χ_Y_; in gray) of the Me_m_YH fragments (Y = C, Si, Ge, N, P, As, O, S, Se; m = 3, 2, 1). The electronegativity of H is χ_H_ = 2.20.^8^ Computed at ZORA-BLYP-D3(BJ)/TZ2P.

We start by analyzing the protonic or hydridic character of the H atom of the studied model Me_m_YH fragments, in which Y is an element belonging to groups 14, 15, and 16 and periods 2, 3, and 4 of the periodic table (Y = C, Si, Ge, N, P, As, O, S, Se, and m = 3, 2, and 1). For this purpose, we analyze the Voronoi deformation density (VDD) charge on the H atom (*Q*_H_) directly bound to atom Y (see Theoretical Methods). In general, we find that Y atoms with an electronegativity higher than H (χ_H_ = 2.20)^8^ cause a charge depletion on H, i.e., a positive VDD charge on H, whereas Y atoms with an electronegativity lower than H result in an accumulation of charge on H, i.e., a negative VDD charge on H. For example, *Q*_H_ is +154 milli-electrons for MeOH and −92 milli-electrons for Me_3_SiH, where the electronegativity of O and Si are χ_O_ = 3.44 and χ_Si_ = 1.90, respectively (Figure 1). Thus, the nature of atom Y greatly influences the charge of the H atom in the Me_m_YH monomer, making them either protonic or hydridic.

We recall that, according to the IUPAC definition,^2^ a hydrogen bond is only formed when the Y atom is more electronegative than H. In other words, the Me_3_SiH, Me_3_GeH, Me_2_PH, and Me_2_AsH fragments with hydridic H should be unable to engage in a hydrogen bond with an electron-rich fragment, i.e., a Lewis base (Figure 1). In the following section, we quantify this statement and explain why hydrogen bonds involving a hydric hydrogen can indeed not be formed.

### On the Nonexistence of Hydridic Hydrogen Bonds

**Figure 2:**
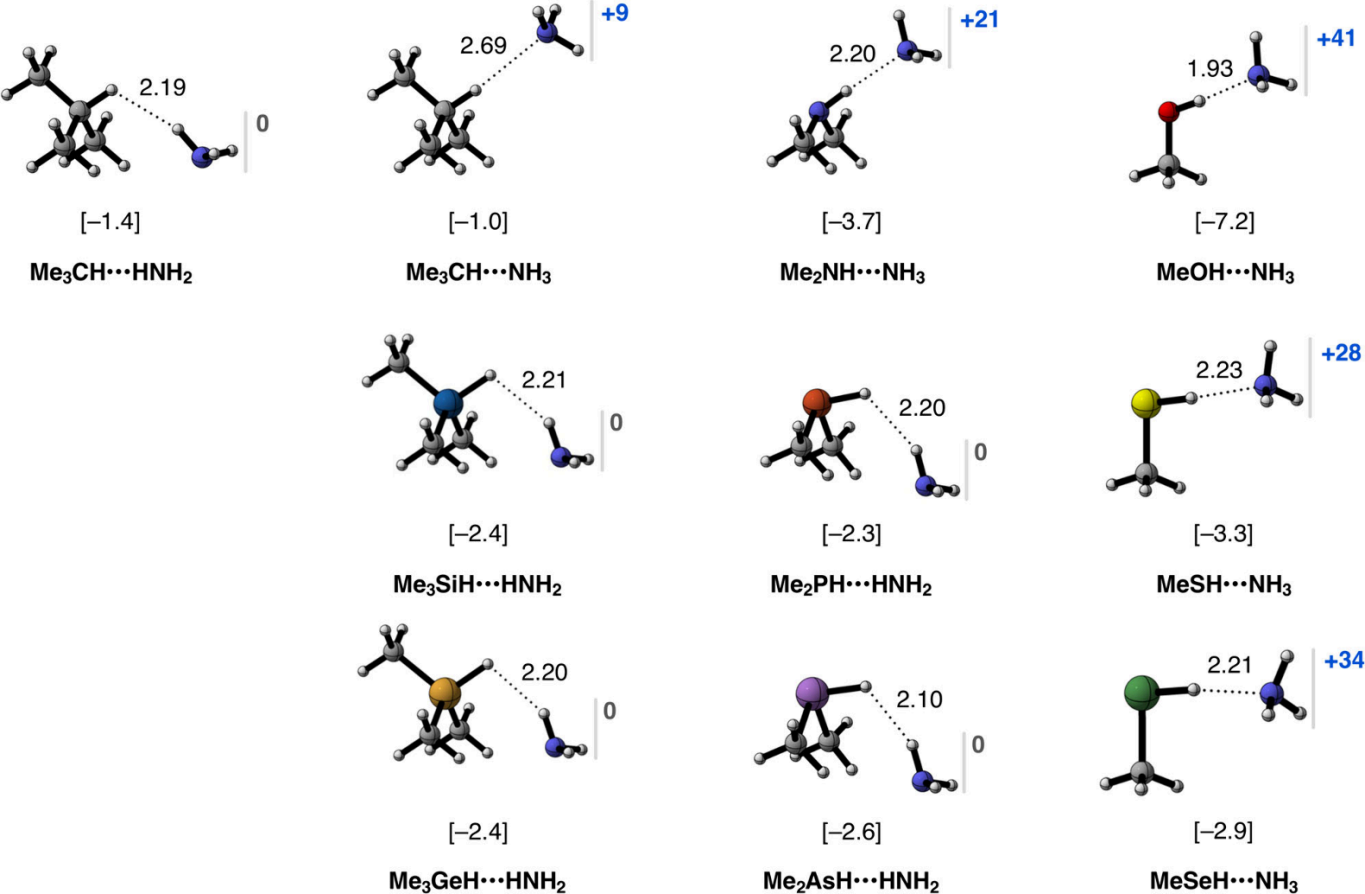
Equilibrium geometries (in Å), electronic bond energies (in kcal mol^–1^; in brackets), and the charge depletion on the NH_3_ fragment (Δ*Q*_NH3_; in milli-electrons; in blue) of the Me_m_YH···NH_3_ hydrogen-bonded complexes (Y = C, N, O, S, Se) and the Me_m_YH···HNH_2_ complexes (Y = C, Si, Ge, P, As). Computed at ZORA-BLYP-D3(BJ)/TZ2P.

To evaluate the ability of protonic and hydridic hydrogens to engage in hydrogen bonding, we study the interaction between the Me_m_YH fragments and NH_3_, a well-known Lewis base and hydrogen-bond acceptor, to form complexes of the type Me_m_YH···NH_3_ (see Scheme 1a). The equilibrium geometries, electronic bond energies Δ*E*, and the charge depletion on the NH_3_ fragment are reported in Figure 2 (see Table S1 for additional data). We find that Me_m_YH···NH_3_ complexes exist only for the Me_m_YH fragments with protonic or close to neutral hydrogens, namely, for Y = C, N, O, S, and Se, in which the most stable and shortest is MeOH···NH_3_, with Δ*E* = −7.2 kcal mol^–1^ and *r*_H···N_ = 1.93 Å, and the least stable and longest is Me_3_CH···NH_3_, with Δ*E* = −1.0 kcal mol^–1^ and *r*_H···N_ = 2.68 Å (Figure 2). The fragments with hydridic hydrogens (Y = Si, Ge, P, As), but also the slightly protonic Me_3_CH, form structurally completely different bonding motifs of the type Me_3_YH···HNH_2_, which are less stable, ranging from Me_2_AsH···HNH_2_, with Δ*E* = −2.6 kcal mol^–1^, to Me_3_CH···HNH_2_, with Δ*E* = −1.4 kcal mol^–1^ (Figure 2).

**Scheme 2:**
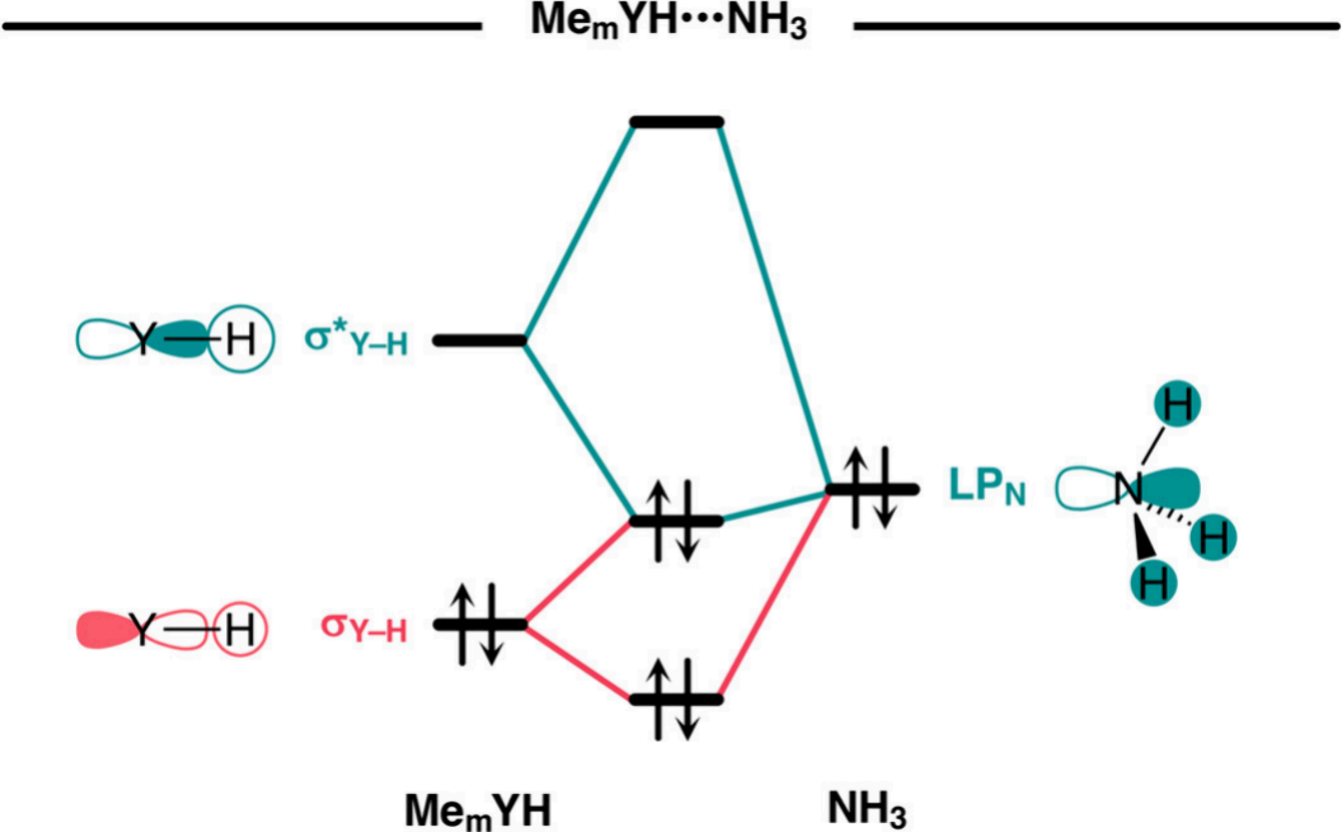
Schematic Molecular Orbital Diagram for the Attractive (Green) and Repulsive (Red) Orbital Interactions in Me_m_YH···NH_3_ (Y = C, N, O, S, Se)

The different bonding motifs in protonic Me_m_YH···NH_3_ versus those in hydridic Me_m_YH···HNH_2_ are accompanied by different bonding mechanisms. In Me_m_YH···NH_3_, we find a depletion of charge on NH_3_ upon binding, i.e., positive Δ*Q*_NH3_, that is, there is charge transfer from NH_3_ into the Me_m_YH fragment (see Figure 2). The charge transfer mechanism from NH_3_ to Me_m_YH is the characteristic covalent component of the hydrogen bond, where the lone pair orbital of NH_3_ (LP_N_) donates charge into the empty σ* Y–H antibonding orbital of Me_m_YH (σ*_Y–H_; see Scheme 2).^3^ As the σ*_Y–H_ becomes more populated, the Y–H bond elongates (see Table S1), resulting in the characteristic redshift in the vibrational frequency associated with the Y–H bond stretching mode. In contrast, there is no charge transfer in the Me_m_YH···HNH_2_ bonding motif involving a hydridic hydrogen, that is, there is no charge depletion or accumulation on NH_3_ and, hence, no hydrogen bond in these complexes (see Figure 2). Next, we explain why hydridic fragments cannot form Me_m_YH···NH_3_ hydrogen-bonded complexes.

Stable hydrogen-bonded complexes are formed because the electrostatic attraction between Y–H^δ+^ and the electron-rich bond acceptor, together with the covalent, charge transfer interaction, overcomes the destabilizing Pauli repulsion between the occupied σ Y–H bonding orbital (σ_Y–H_) and the occupied orbitals of the bond acceptor (see Scheme 2).^3^ Protonic hydrogens are depleted of electron density, which exposes their positively charged nucleus to the electron density of an incoming electron-rich fragment, resulting in a stronger electrostatic attraction. On the other hand, hydridic hydrogens have an excess of electron density that: (i) screens their positively charged nucleus, causing a weaker electrostatic attraction between Y–H^δ−^ and the hydrogen-bond acceptor; and (ii) increases the size of σ_Y–H_ on the H atom, resulting in a more destabilizing Pauli repulsion between σ_Y–H_ and the occupied orbitals of the bond acceptor. As a result, hydrogen-bonded complexes involving a hydridic hydrogen are not formed because the attractive electrostatic and charge transfer interactions are not strong enough to overcome the repulsive wall arising from the Pauli repulsion. These findings emerge from our energy decomposition and quantitative KS-MO analyses that will be explained in detail in the next section.

**Figure 3:**
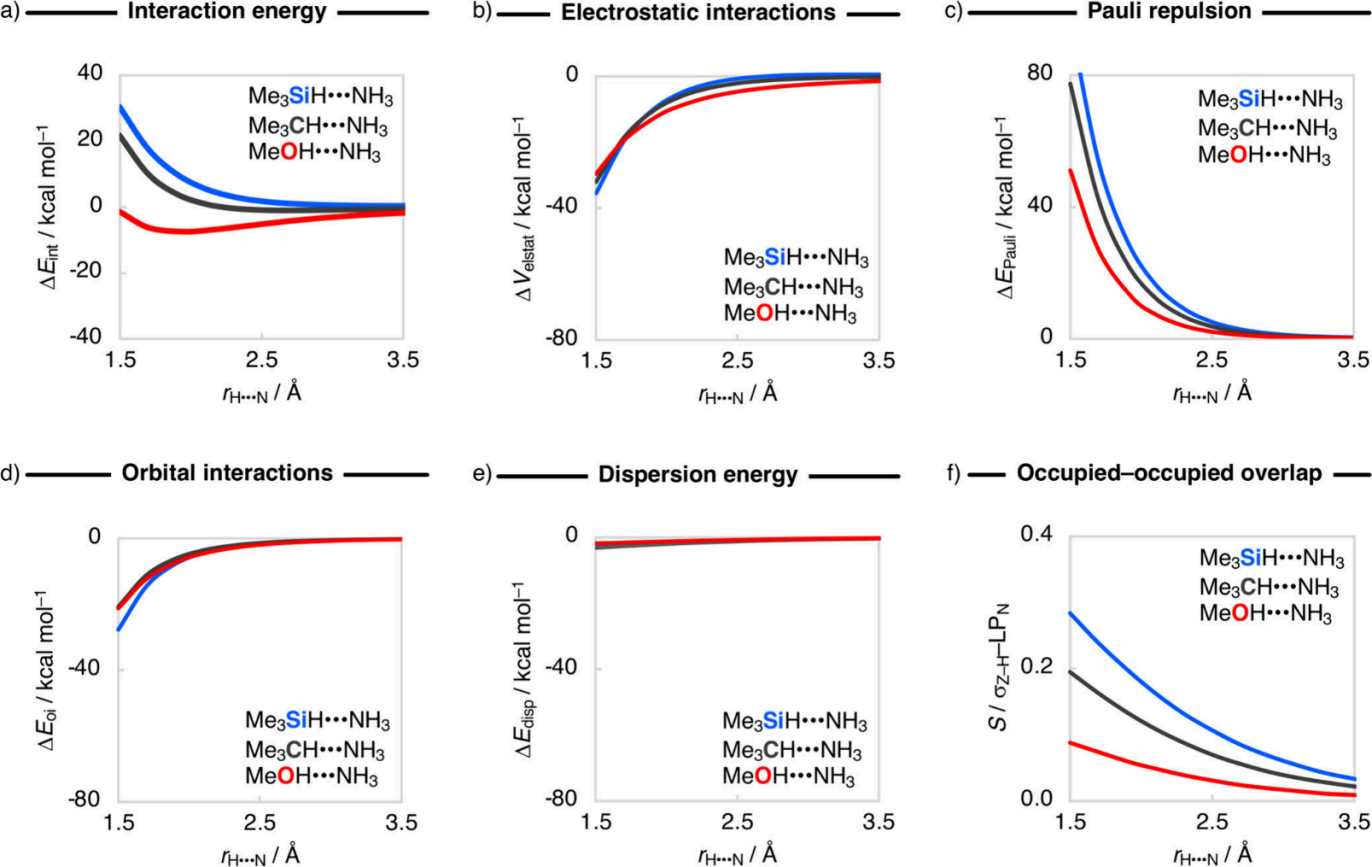
Energy decomposition analysis (in kcal mol^–1^) and the repulsive occupied–occupied overlap (*S*) as a function of the H···N bond distance for the representative Me_3_SiH···NH_3_, Me_3_CH···NH_3_, and MeOH···NH_3_ complexes. Computed at ZORA-BLYP-D3(BJ)/TZ2P.

First, we analyze the bonding mechanism of the representative MeOH···NH_3_, Me_3_CH···NH_3_, and Me_3_SiH···NH_3_ hydrogen-bonded complexes using our canonical energy decomposition analysis (EDA)^9^ as a function of the H···N bond distance (*r*_H···N_), while keeping the geometries of the fragments frozen to the equilibrium geometries of the complexes (Figure 3; see Theoretical Methods). The Me_3_SiH···NH_3_ complex is unbound; therefore, in this case, we have the Me_3_SiH and NH_3_ fragments approach each other along the *r*_H···N_ in their respective equilibrium geometries.

**Figure 4:**
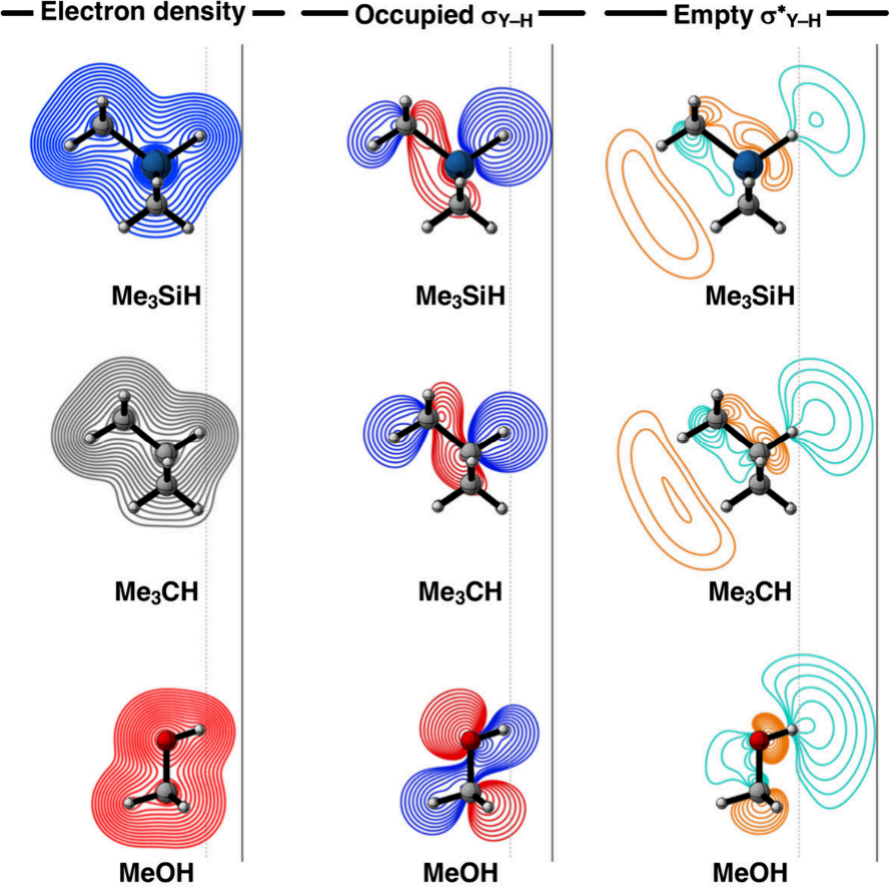
Electron density, occupied σ Y–H bonding orbital, and empty σ* Y–H antibonding orbital (contour plots from 0.9 to 0.03 au) for the representative Me_3_SiH, Me_3_CH, and MeOH fragments. Computed at ZORA-BLYP-D3(BJ)/TZ2P.

The interaction energy curve, Δ*E*_int_(*r*_H···N_), goes from stabilizing, i.e., binding, for MeOH···NH_3_ to weakly stabilizing for Me_3_CH···NH_3_ to destabilizing, *i.e*, unbound, for Me_3_SiH···NH_3_, reflecting the trends in hydrogen-bond stability of the complexes (Figure 3a). This trend is a direct result of the Pauli repulsion, Δ*E*_Pauli_(*r*_H···N_), which becomes significantly more destabilizing from MeOH···NH_3_ to Me_3_CH···NH_3_ to Me_3_SiH···NH_3_ (Figure 3c). Along this series, Y becomes less electronegative than H, which significantly affects the size of the occupied σ_Y–H_ orbital on the H atom. The orbital amplitude on the H atom substantially increases along MeOH to Me_3_CH to Me_3_SiH (see Figure 4), causing the repulsive orbital overlap between the σ_Y–H_ orbital of Me_m_YH and the LP_N_ orbital of NH_3_ to increase along MeOH···NH_3_, Me_3_CH···NH_3_, and Me_3_SiH···NH_3_ and, thus, leading to more build-up of Pauli repulsion upon bond formation (see Figure 3f). Note that the reduced Pauli repulsion is also the reason why MeOH···NH_3_ can form a shorter hydrogen bond compared to the other complexes (Figure 3). In addition, the electrostatic interactions curve, Δ*V*_elstat_(*r*_H···N_), also becomes less stabilizing along MeOH···NH_3_, Me_3_CH···NH_3_, and Me_3_SiH···NH_3_, especially around the equilibrium hydrogen-bond bond length (Figure 3b). This is a direct consequence of the decreasing electronegativity of Y along O, C, and Si, which makes the donating H atom less positively (or more negatively) charged along the same series, leading to a weaker electrostatic attraction between Me_m_YH and the electron-rich NH_3_ fragment. In summary, a hydridic hydrogen atom causes the Me_m_YH···NH_3_ complexes to experience a substantial build-up in steric repulsion and a weakening of electrostatic attraction, resulting in the nonexistence of hydridic hydrogen bonds in Me_m_YH···NH_3_.

### Hydridic Hydrogens as Bond Acceptors

**Figure 5:**
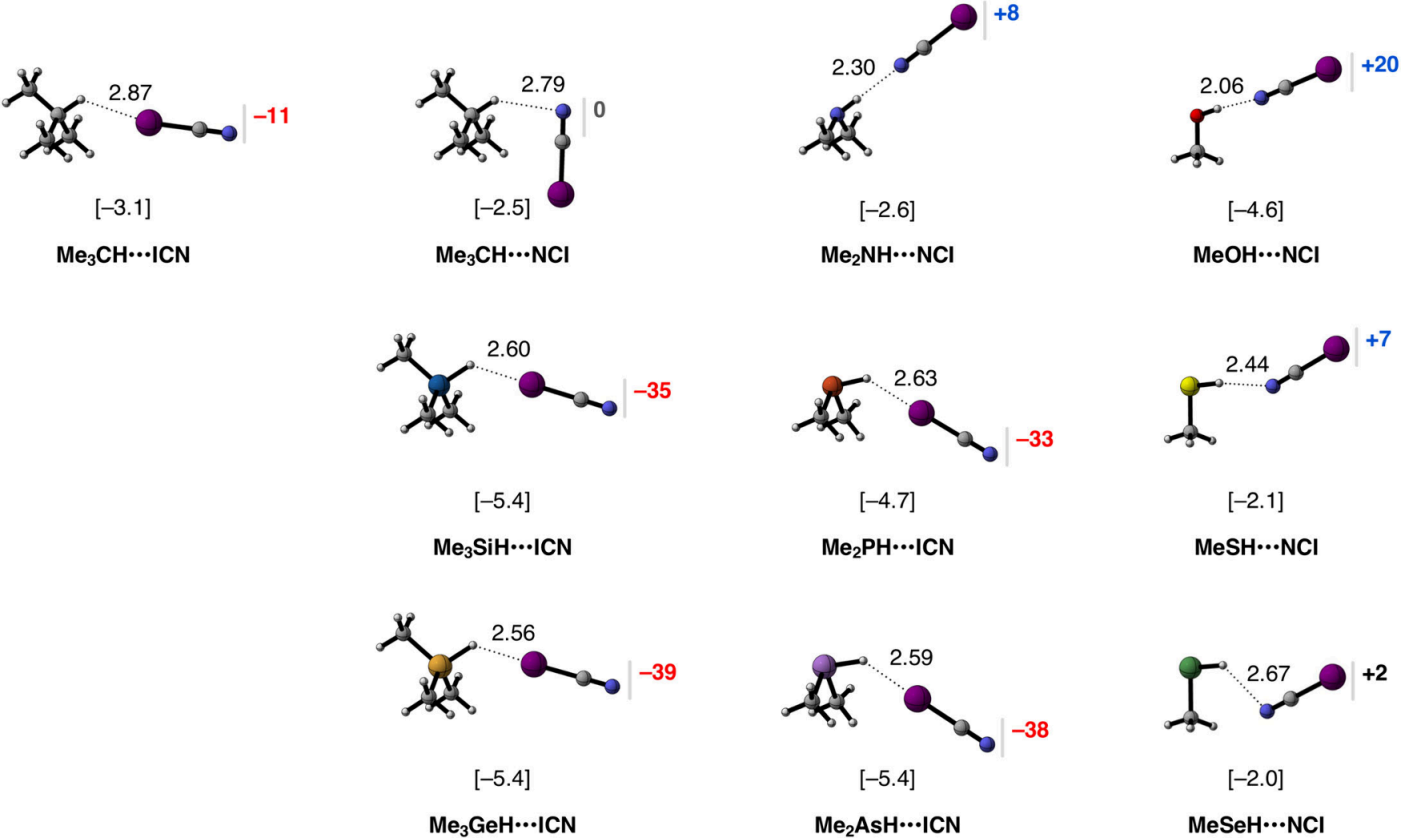
Equilibrium geometries (in Å), electronic bond energies (in kcal mol^–1^; in brackets), and the charge accumulation (red) and depletion (blue) on the ICN fragment (in milli-electrons) of the Me_m_YH···NCI complexes (Y = C, N, O, S, Se) and Me_m_YH···ICN (Y = C, Si, Ge, P, As). Computed at ZORA-BLYP-D3(BJ)/TZ2P.

We have shown that hydridic hydrogens are unable to engage in hydrogen bonds with electron-rich molecules like NH_3_. In this section, we address the question: can hydrogen bonding be the most dominant interaction when a hydridic hydrogen engages in an intermolecular interaction with electron-poor halogen-bond donors as proposed by Civiš, Hobza, and co-workers?^6^ To answer this question, we study the bonding mechanism for the interaction between Me_m_YH and ICN, where ICN can form a hydrogen bond with the nitrogen side and a halogen bond with the iodine side,^10^ according to the IUPAC definitions of hydrogen bonds^2^ and halogen bonds^10a^ (see Scheme 1). The equilibrium geometries, electronic bond energies Δ*E*, and the charge rearrangement in the ICN fragment are reported in Figure 5.

Similar to Me_m_YH···NH_3_, the Me_m_YH fragments involving protonic and close to neutral hydrogens (Y = C, N, O, S, Se) form regular Y–H^δ+^···N^δ−^ hydrogen bonds, in which charge flows into the hydrogen-bond donor Me_m_Y–H, i.e., positive Δ*Q*_ICN_ (Figure 5). This bond has a strength of up to 4.6 kcal mol^–1^ for MeOH···NCI (Figure 5). On the other hand, the Me_m_YH fragments involving a hydridic hydrogen (Y = Si, Ge, P, As) form regular H^δ−^···I^δ+^–C halogen bonds, in which charge flows out of the halogen-bond acceptor Me_m_Y–H into the halogen-bond donor I–CN, i.e., negative Δ*Q*_ICN_ (Figure 5). There is one intermediate situation, namely, the Me_3_CH fragment, which shows both Me_3_CH···NCI and Me_3_CH···ICN bonding modes. As will become clear, this change in bonding mode occurs because we go from protonic Me_m_YH···NCI hydrogen bonds to Me_m_YH···ICN halogen bonds, *not* hydridic Me_m_YH···ICN hydrogen bonds, in line with the current IUPAC definitions of hydrogen bonds^2^ and halogen bonds.^10a^

**Scheme 3:**
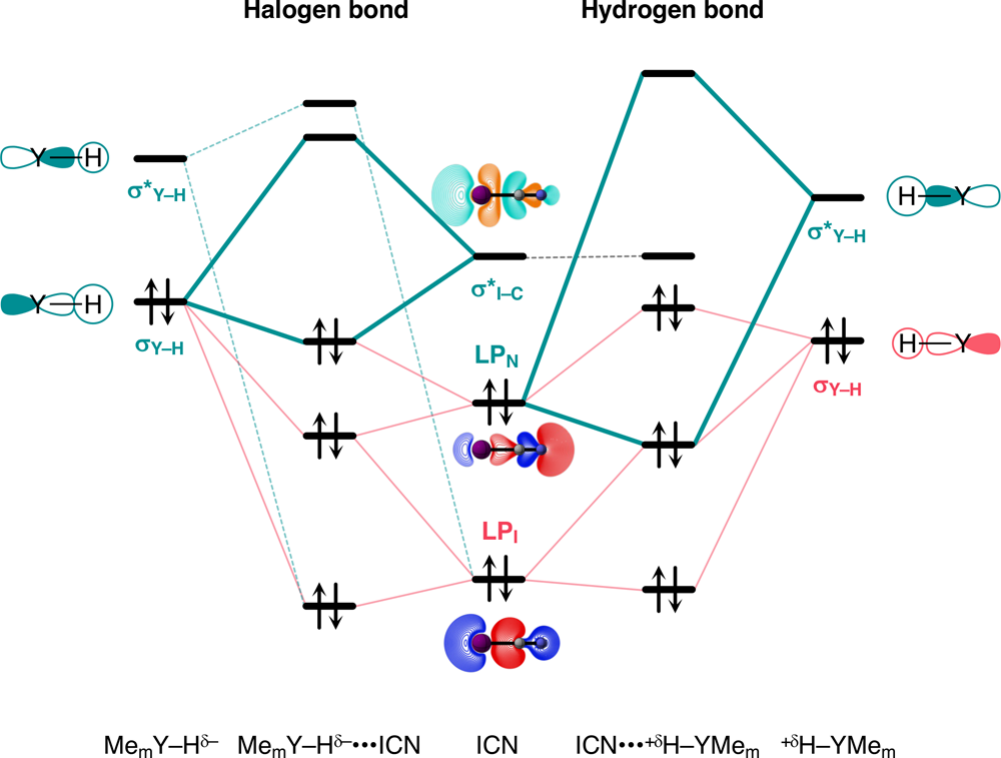
Schematic Molecular Orbital Diagram (Contour Plots from 0.9 to 0.03 a.u.) for the Attractive (Green) and Repulsive (Red) Orbital Interactions in the Halogen-Bonded Me_m_YH···ICN and Hydrogen-Bonded Me_m_YH···NCI Complexes (Computed at ZORA-BLYP-D3(BJ)/TZ2P)

The bonding mechanism in Me_m_YH···NCI involves a regular Y–H^δ+^···N^δ−^ hydrogen bond and hence is similar to that of the Me_m_YH···NH_3_ complexes (*vide supra*). The ICN fragment acts as a hydrogen-bond acceptor on the N side through which the LP_N_ orbital of the ICN fragment donates charge into the empty σ*_Y–H_ orbital of Me_m_YH fragments involving protonic hydrogens (Scheme 3, right side). However, as explained in the previous section, this donor–acceptor interaction is not strong enough to overcome the buildup of steric repulsion when involving Me_m_YH fragments with hydridic hydrogens and, thus, Me_m_YH···NCI complexes cannot be found for Y = Si, Ge, P, and As.

The hydridic Me_m_YH···ICN complexes are constructed via a different bonding mechanism, namely, a halogen-bond,^3b,11^ which is characterized by the covalent, donor–acceptor interaction between the occupied σ_Y–H_ orbital of Me_m_YH and the empty σ* I–C antibonding orbital of ICN (σ*_I–C_) (Scheme 3, left side). Consequently, upon binding, the charge flows from Me_m_YH into the ICN fragment, as indicated by the negative Δ*Q*_ICN_ (see Figure 5). Note that the hydrogen-bonding and halogen bonding mechanisms go with charge transfer in opposite directions; that is, Δ*Q*_ICN_ is positive for the former and negative for the latter. Therefore, the negative Δ*Q*_ICN_ is clear evidence that there is no hydrogen-bonding mechanism that is able to overcome the halogen bond in the Me_m_YH···ICN complexes. This is because ICN is a poor hydrogen-bond acceptor, i.e., weak Lewis base, on the I side due to its very low-lying I lone-pair (LP_I_) orbital (see Scheme 3).

**Figure 6:**
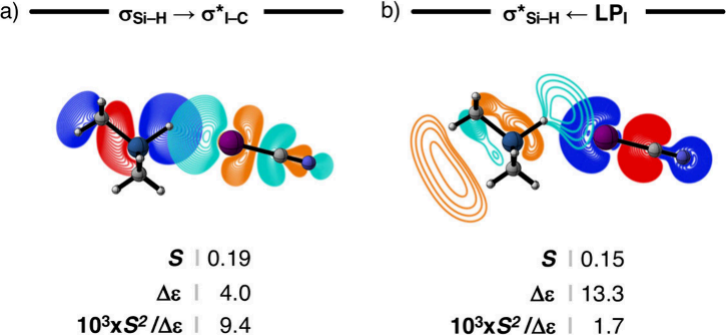
Donor–acceptor orbital overlap (*S*), energy gap (Δε, in eV), and the orbital stabilization for a) the σ_Si–H_ → σ*_I–C_ halogen bond and b) the σ*_Si–H_ ← LP_I_ hydrogen bond in the representative Me_3_SiH···ICN complex (contour plots from 0.9 to 0.03 au). Computed at ZORA-BLYP-D3(BJ)/TZ2P.

The magnitude of the various pairwise donor–acceptor orbital interactions is approximately proportional to their orbital overlap squared (*S*^2^) divided by the orbital energy gap (Δε). For Me_3_SiH···ICN, the *S* and Δε for the σ_Y–H_ → σ*_I–C_ halogen-bond interaction are 0.19 and 4.0 eV, respectively, and its associated *S*^2^/Δε is 9.4 × 10^3^ (see Figure 6 and Table S5 for all the Me_m_YH···ICN complexes). The LP_I_ orbital of ICN has a large amplitude on the I side and overlaps with the empty σ*_Y–H_ of Me_3_SiH, in which *S* is 0.15 (Figure 6). However, this σ*_Y–H_ ← LP_I_ hydrogen-bond interaction is significantly weaker due to a large Δε of 13.3 eV, leading to an *S*^2^/Δε of only 1.7 × 10^3^, which is about five times weaker than that of the σ_Y–H_ → σ*_I–C_ halogen-bond interaction (Figure 6). Therefore, the magnitude of the hydrogen-bonding mechanism is negligible and dominated by the halogen bond in Me_3_SiH···ICN. For this reason, Me_3_SiH···ICN and the other Me_m_YH···ICN complexes involving hydridic hydrogens should be seen as halogen-bonded, *not* hydridic hydrogen-bonded, complexes.

### The Redshift

The analyzed Me_m_YH···NH_3_ hydrogen-bonded systems feature the characteristic Y–H bond elongation and, thus, the redshift in the Y–H bond stretching frequency. For example, in the series of MeOH···NH_3_, MeSH···NH_3_, and MeSeH···NH_3_, the Y–H bond elongates by 0.016 Å, 0.010 Å, and 0.012 Å, and the symmetric Y–H bond stretching frequency in the complexes decreases by −316 cm^–1^, −125 cm^–1^, and −134 cm^–1^, respectively, in respect to the isolated Me_m_YH monomers (Table 1). The Y–H bond elongation in hydrogen-bond donors is the manifestation of the covalent nature of hydrogen bonds, that is, it is caused by the donation of charge into the empty σ*_Y–H_ orbital via donor–acceptor interactions.^3b,4^ In the series of MeOH···NH_3_, MeSH···NH_3_, and MeSeH···NH_3_, there is a depletion of charge in the NH_3_ fragment, i.e., positive Δ*Q*_NH3_, while the population in empty σ*_Y–H_ orbital increases to 0.04 0.03, and 0.03 electrons along the same series.

**Table 1:** Y–H Bond Elongation (in Å), Change in the Y–H Stretching Frequency (in cm^–1^), Charge in the Voronoi Deformation Density Charge on the NH_3_ or ICN Fragments (in Milli-electrons), and Populations in the Filled σ_Y–H_ and Empty σ*_Y–H_ Orbitals for Representative Me_m_YH···NH_3_ (Y = O, S, Se) and Me_m_YH···ICN (Y = C, Si, Ge) Complexes (Computed at ZORA-BLYP-D3(BJ)/TZ2P^a^)

**Complex**	**Δ*****r***_**Y–H**_	**Δυ**_**Y–H**_	**Δ*****Q***_**NH3/ICN**_	**Pop.σ**_**Y–H**_	Pop.σ*_Y–H_
**MeOH···NH**_**3**_	0.016	–316	+41	2.00	0.04
**MeSH···NH**_**3**_	0.010	–125	+28	2.00	0.03
**MeSeH···NH**_**3**_	0.012	–134	+34	2.00	0.03
					
**Me**_**3**_**CH···ICN**	0.003	–47	–11	1.99	0.00
**Me**_**3**_**SiH···ICN**	0.014	–87	–35	1.94	0.00
**Me**_**3**_**GeH···ICN**	0.019	–98	–39	1.93	0.01

a See complete data in Tables S1–S4 in the Supporting Information.

In the halogen-bonded Me_m_YH···ICN complexes, we find that the Y–H bond also elongates and, thus, the Y–H bond stretching frequency redshifts. For example, in the series of Me_3_CH···ICN, Me_3_SiH···ICN, and Me_3_GeH···ICN, the Y–H bond elongates by 0.003 Å, 0.014 Å, and 0.019 Å, and the symmetric Y–H bond stretching frequency in the halogen-bond acceptors decreases by −47 cm^–1^, −87 cm^–1^, and −98 cm^–1^, respectively (Table 1). However, since the hydrogen-bonding mechanism is negligible in the Me_m_YH···ICN complexes (*vide supra*), the population in the empty σ*_Y–H_ orbital does not significantly change and, therefore, cannot be responsible for the Y–H bond elongation in these complexes (Table 1). In fact, the redshift in the Me_m_YH halogen-bond acceptors is a consequence of their Lewis basicity. In the previous section, we showed that the halogen-bond donor–acceptor mechanism in the Me_m_YH···ICN complexes is the donation of charge from the filled σ_Y–H_ orbital of Me_m_YH into the empty σ*_I–C_ orbital of ICN. For example, in the series of Me_3_CH···ICN, Me_3_SiH···ICN, and Me_3_GeH···ICN, the population in the filled σ_Y–H_ orbital is reduced to 1.99 electrons, 1.94 electrons, and 1.94 electrons respectively, while there is charge accumulation in the ICN fragment, i.e., negative Δ*Q*_ICN_ (Table 1). Consequently, the Y–H bond becomes longer and, thus, redshifts, as the σ_Y–H_ bonding orbital loses electrons due to the halogen bond. Therefore, the redshift in the Y–H bond stretching frequency should also be expected in situations when hydridic Y–H^δ−^ fragments behave as a Lewis base and hence is *not* an exclusive feature of hydrogen-bond donors.

A similar phenomenon is found when the hydridic hydrogen of Me_3_SiH engages in a stabilizing intermolecular interaction with the different electron-poor molecules studied by Civiš, Hobza, and co-workers,^6^ that is, ICF_3_, BrCN, S(CN)_2_, P(CN)_3_, and K^+^. In all these complexes, the Si–H bond elongates and redshifts upon complex formation. However, this is *not* because Me_3_SiH behaves a hydrogen-bond donor, but as a bond acceptor. We find that there is charge transfer from Me_3_SiH into the electron-poor fragments and the population in the filled σ Si–H bonding orbital is reduced, which, in fact, characterizes different intermolecular interactions, e.g., halogen bonds, chalcogen bonds, and pnictogen-bonds^11^ (see Table S6; see addition analyses on palladium hydride, boron hydride, beryllium hydride, and lithium hydride in Tables S7 and S8). A bonding motif in which a hydridic hydrogen acts as the acceptor of a hydrogen bond exists in the particular form of dihydrogen bonds (DHB); this has been described elsewhere.^7^ Thus, we have presented clear evidence that the interaction between the hydridic hydrogen of Me_3_YH and an electron-poor molecule should not be seen as any kind of hydrogen bonding but instead as a different intermolecular interaction named after the nature of the electron-poor bond donor.

## Conclusions

We have quantum chemically analyzed the bonding mechanism of a series of Me_m_YH···NH_3_ and Me_m_YH···ICN complexes, in which Y = C, Si, Ge, N, P, As, O, S, Se and m = 3, 2, 1, using quantitative Kohn–Sham molecular orbital (KS-MO) theory. We showed that Me_m_Y–H^δ+^ fragments involving protonic hydrogens (i.e., Y = C, N, O, S, Se) form regular Y–H^δ+^···N^δ−^ hydrogen bonds, whose stability is due to the H^δ+^···N^δ−^ electrostatic attraction and the charge transfer from the hydrogen-bond acceptor NH_3_ into the hydrogen-bond donor Me_m_Y–H^δ+^. The Me_m_Y–H^δ−^ fragments involving hydridic hydrogens (i.e., Y = Si, Ge, P, As) are unable to act as hydrogen-bond donors and, instead, behave as Lewis bases, donating charge into electron-poor fragments, such as ICN, forming a H^δ−^···I^δ+^–C halogen bond. Therefore, our findings do not support the proposal to change the IUPAC definition of hydrogen bonds to include molecules with partially negatively charged H as hydrogen-bond donors.^6^

Regular hydrogen bonds, as in Me_m_Y–H^δ+^···NH_3_, involve charge transfer into the empty σ* Y–H antibonding orbital of the hydrogen-bond donor fragment. This hydrogen-bond donor–acceptor interaction is negligible in the Me_m_Y–H^δ−^···ICN complexes, and the charge transfer goes in the opposite direction from the occupied σ Y–H bonding orbital into the ICN fragment. This is because ICN is a poor electron donor on the I side, which leads to a weaker hydrogen-bond donor–acceptor interaction in Me_m_Y–H^δ−^···ICN. In turn, the covalent component in Me_m_Y–H^δ−^···ICN is dominated by the halogen-bond donor–acceptor interaction between the occupied σ Y–H bonding orbital and the empty σ* I–C antibonding orbital of ICN. Therefore, the Me_m_Y–H^δ−^ fragments are halogen-bond acceptors, *not* hydrogen-bond donors.

The accumulation of charge in the empty σ* Y–H antibonding orbital causes the Y–H bond elongation and, thus, the typical redshift in the of the Y–H stretching frequency for regular Me_m_Y–H^δ+^···NH_3_ hydrogen bonds. However, the elongation the Y–H bond can also be caused by other factors, e.g., the depletion of charge in the occupied σ Y–H bonding orbital in halogen-bonded Me_m_Y–H^δ−^···ICN complexes. Therefore, the redshift in the Y–H stretching frequency should not be used as the unique but as one of the diagnostics to characterize hydrogen bonds.

## Theoretical Methods

### Computational Details

All calculations were carried out using the Amsterdam Density Functional (ADF) 2023.101 program.^12^ All stationary points and energies were obtained using relativistic, dispersion-corrected density functional theory at ZORA-BLYP-D3(BJ)/TZ2P (see Tables S9, S10, and S11 in the Supporting Information for the Cartesian coordinates). This approach comprises the BLYP level of the generalized gradient approximation (GGA); the exchange functional developed by Becke (B), and the GGA correlation functional developed by Lee, Yang, and Parr (LYP).^13^ The empirical DFT-D3(BJ) correction developed by Grimme and co-workers,^14^ which contains the damping function proposed by Becke and Johnson,^15^ is used to account for nonlocal dispersion interactions. Scalar relativistic effects are accounted for using the zeroth-order regular approximation (ZORA).^16^ This level has been proven to accurately describe weak interactions.^17^ Molecular orbitals (MO) were expanded into a large, uncontracted set of Slater-type orbitals (STOs) containing diffuse functions: TZ2P.^18^ The basis set is of triple-ζ quality augmented with polarization functions, i.e., one 2p and one 3d set on H; one 3d and one 4f set on C, N, O, Si, P, S; one 4d and one 4f set on Ge, As, Se; one 5d and one 4f set on I. All electrons were included in the variational process; i.e., no frozen core approximation was applied. The accuracies of both the Zlm fitting scheme^19a^ and the Becke integration grid^19b^ were set to ‘EXCELLENT’.

### Bond Analyses

Insight into the bonding mechanism is obtained by analyzing the intermolecular interaction between Me_m_YH (Y = C, Si, Ge, N, P, As, O, S, Se) and the NH_3_ or ICN fragments using the activation strain model,^9^ which is a fragment-based approach to understanding the energy profile associated with a chemical process in terms of the original reactants. Thus, the total bond energy Δ*E* is decomposed into the strain energy Δ*E*_strain_, which is associated with the geometrical deformation of the individual reactants as the process takes place, plus the actual interaction energy Δ*E*_int_ between the deformed reactants (eq 4).
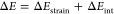
4The interaction energy Δ*E*_int_ between the deformed reactants is further analyzed in the conceptual framework provided by the quantitative Kohn–Sham MO model.^9^ To this end, it is decomposed into physically meaningful terms, using a quantitative energy decomposition analysis (EDA) as implemented in ADF (eq 5).^9^ The analyses are done as a function of the H···N (*r*_H···N_) or H···I (*r*_H···I_) bond distances along the range of 1.5 to 3.5 Å. Since Δ*E*_strain_ is negligible (see Tables S1 and S3), we performed the analyses starting from the equilibrium geometry of the complex while keeping all other geometrical parameters frozen. Values in the equilibrium geometries are shown in Tables S2 and S4.
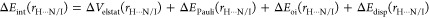
5Δ*V*_elstat_ is the classical Coulomb interaction between the unperturbed charge distributions of the deformed reactants which is usually stabilizing and comprises four components: (i) the electron–electron electrostatic repulsion between the electron densities of fragments 1 and 2, Δ*V*_elstat,ρ_1_ρ_2__; (ii) the nuclei–electron electrostatic attraction between the nuclei of fragment 1 and the electron density of fragment 2, Δ*V*_elstat,*n*_1_ρ_2__; (iii) the electron–nuclei electrostatic attraction between the electron density of fragment 1 and the nuclei of fragment 2, Δ*V*_elstat,ρ_1_*n*_2__; and (iv) the nuclei–nuclei electrostatic repulsion between the nuclei of fragments 1 and 2, Δ*V*_elstat,*n*_1_*n*_2__.

The Pauli repulsion energy (Δ*E*_Pauli_) comprises the destabilizing interactions between occupied orbitals on either fragment (more precisely, between same-spin occupied spinorbitals on either fragment) and arises from the antisymmetrization of the Hartree wave function due to the Pauli principle. The orbital-interaction energy (Δ*E*_oi_) accounts for charge transfer, that is, the interaction between occupied orbitals of one fragment with unoccupied orbitals of the other fragment, including the interactions of the highest occupied and lowest unoccupied MOs (HOMO–LUMO), and polarization, that is, empty–occupied orbital mixing on one fragment, due to the presence of another fragment. Δ*E*_disp_ accounts for the empirical dispersion corrections as introduced by Grimme et al.^14^ To facilitate the analyses, the ASM and EDA were performed using the PyFrag 2019 program.^20^

### Voronoi Deformation Density (VDD) Charge Analysis

The Voronoi Deformation Density (VDD) charge analysis allows for the quantification of the flow of electronic charge as a consequence of chemical-bond formation.^21^ In our model Me_m_YH fragments, the VDD atomic charge of atom H directly bound to Y (*Q*_H_) is computed by the spatial integration of the deformation density over the Voronoi cell of atom H, which is the space defined by the bond midplanes on and perpendicular to all bond axes between atom H and its neighboring atoms (eq 1).
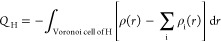
1Herein, the deformation density Δρ(*r*) = [ρ(*r*) – ∑_i_ρ_i_(*r*)] is the density change going from a superposition of the original atomic densities at the positions of the molecule to the actual density of that molecule. This atomic or so-called promolecular density is defined as the sum of the (spherically averaged) ground-state atomic densities ∑_i_ρ_i_(r). This is the fictitious state in which the charge density has not been affected by chemical bonding and in which all atoms have zero charge. Then, *Q*_H_ represents the amount of charge that, due to chemical bonding, flows from a position closer to another nucleus to a position closer to the nucleus of H (*Q*_H_ < 0), or from a position closer to the nucleus of H to a position closer to another nucleus (*Q*_H_ > 0).

Besides the above regular VDD atomic charges relative to noninteracting neutral atoms, the VDD method allows for the analysis of changes in atomic charges (Δ*Q*), i.e., charge-density rearrangements, caused by interactions between molecular fragments (eq 2).
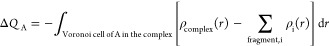
2Equation 2 defines the charge rearrangements in an atom A of a fragment *i* (Δ*Q*_A_) in the final density of the overall complex ρ_complex_(*r*) relative to the sum of the initial molecular fragment densities ∑_fragment,i_ρ_i_(*r*)_i_. This reveals how the interactions between the molecular fragments affect the electron density distribution in atom A of fragment *i*. The sum of the Δ*Q*_A_ of all atoms in a fragment *i* gives the amount of charge transfer into (Δ*Q*_fragment,i_ < 0) or out of (Δ*Q*_fragment,i_ > 0) the Voronoi cell of a fragment *i* due to the interaction between the molecular fragments (eq 3).
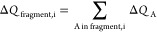
3

## Supporting Information Available

Activation strain, energy decomposition, and VDD analyses; and Cartesian coordinates of all stationary points. (PDF)
