# Supplementary material for: “Hydridic Hydrogen-Bond Donors” Are Not Hydrogen-Bond Donors
Source: J Am Chem Soc. 2024 Sep 3;146(37):25701–9. doi: 10.1021/jacs.4c07821 (PMC11421000; doi:10.1021/jacs.4c07821)
Supplement: Supplementary file 1 — ja4c07821_si_001.pdf [file ja4c07821_si_001.pdf]

Supporting Information for

"Hydridic Hydrogen-Bond Donors" Are Not Hydrogen-Bond Donors

Lucas de Azevedo Santos,[a] Pascal Vermeeren,[a] F. Matthias Bickelhaupt[a,b,c]* and Célia Fonseca Guerra[a]*

[a] Department of Chemistry and Pharmaceutical Sciences, AIMMS, Vrije Universiteit Amsterdam, De Boelelaan 1108, 1081 HZ Amsterdam, The Netherlands.
Email: f.m.bickelhaupt@vu.nl, c.fonsecaguerra@vu.nl

[b] Institute for Molecules and Materials, Radboud University, Heyendaalseweg 135, 6525 AJ Nijmegen, The Netherlands.

[c] Department of Chemical Sciences, University of Johannesburg, Auckland Park, Johannesburg 2006, South Africa.

Table of Contents

Table S1. Activation strain analysis (in kcal mol^{-1}), bond lengths (in Å), change in the Y–H stretching frequency (in cm^{-1}) and change in the Voronoi deformation density charges on the NH_3 fragment for the $Me_mYH•••NH_3$ and $Me_mYH•••HNH_2$ complexes (Y = C, Si, Ge, N, P, As, O, S, Se; m = 3, 2, 1) at their equilibrium geometries. Computed at ZORA-BLYP-D3(BJ)/TZ2P. **S3**

Table S2. Energy decomposition analysis (in kcal mol^{-1}) and populations (in electrons) in the empty σ^*_{Y-H} orbital for the $Me_mYH•••NH_3$ and $Me_mYH•••HNH_2$ complexes (Y = C, Si, Ge, N, P, As, O, S, Se; m = 3, 2, 1). Computed at ZORA-BLYP-D3(BJ)/TZ2P. **S3**

Table S3. Activation strain analysis (in kcal mol^{-1}), bond lengths (in Å), change in the Y–H stretching frequency (in cm^{-1}), and change in the Voronoi deformation density charges on the ICN fragment for the $Me_mYH•••NCI$ and $Me_mYH•••ICN$ complexes (Y = C, Si, Ge, N, P, As, O, S, Se; m = 3, 2, 1) at their equilibrium geometries. Computed at ZORA-BLYP-D3(BJ)/TZ2P. **S4**

Table S4. Energy decomposition analysis (in kcal mol^{-1}) and populations (in electrons) in the occupied σ_{Y-H} and in the empty σ^*_{Y-H} and σ^*_{I-C} orbitals for the $Me_mYH•••NCI$ and $Me_mYH•••ICN$ complexes (Y = C, Si, Ge, N, P, As, O, S, Se; m = 3, 2, 1) at their equilibrium geometries. Computed at ZORA-BLYP-D3(BJ)/TZ2P. **S4**

Table S5. HOMO–LUMO overlap, orbital energies (in eV), and magnitude of donor–acceptor interactions associated with the hydrogen and halogen bonds in the $Me_mYH•••NH_3$, $Me_mYH•••NCI$, and $Me_mYH•••ICN$ complexes (Y = C, Si, Ge, N, P, As, O, S, Se; m = 3, 2, 1). Computed at ZORA-BLYP-D3(BJ)/TZ2P. **S5**

Table S6. Activation strain and energy decomposition analyses (in kcal mol^{-1}), change in the Voronoi deformation density charge on the Me_3SiH fragments (in milli-electrons), and populations (in electrons) in the occupied σ_{Y-H} orbitals upon complexation with different Lewis acids. Computed at ZORA-BLYP-D3(BJ)/TZ2P. **S5**

Table S7. Activation strain analysis (in kcal mol^{-1}), bond lengths (in Å), Voronoi deformation density charge on the H atom of the halogen-bond acceptor fragments in their equilibrium geometries (in milli-electrons), change in the Voronoi deformation density charges on the ICN fragment, and change in the Y–H stretching frequency (in cm^{-1}) for halogen-bonded systems with palladium hydride, boron hydride, beryllium hydride, and lithium hydride. Computed at ZORA-BLYP-D3(BJ)/TZ2P. **S6**

Table S8. Energy decomposition analysis (in kcal mol^{-1}) and populations (in electrons) in the occupied σ_{Y-H} orbital for halogen-bonded systems with palladium hydride, boron hydride, beryllium hydride, and lithium hydride. Computed at ZORA-BLYP-D3(BJ)/TZ2P. **S6**

Table S9. Cartesian coordinates (in Å), ADF total electronic energies (in kcal mol^{-1}), the Y–H stretching frequency (in cm^{-1}), and the number of imaginary vibrational frequencies for the Me_mYH, NH_3, and ICN fragments. Computed at ZORA-BLYP-D3(BJ)/QZ4P. **S7**

Table S10. Cartesian coordinates (in Å), ADF total electronic energies (in kcal mol^{-1}), the Y–H stretching frequency (in cm^{-1}), and the number of imaginary vibrational frequencies for the $Me_mYH\cdots NH_3$ and $Me_mYH\cdots HNH_2$ complexes. Computed at ZORA-BLYP-D3(BJ)/QZ4P. **S10**

Table S11. Cartesian coordinates (in Å), ADF total electronic energies (in kcal mol^{-1}), the Y–H stretching frequency (in cm^{-1}), and the number of imaginary vibrational frequencies for $Me_mYH\cdots NCI$ and $Me_mYH\cdots ICN$ complexes. Computed at ZORA-BLYP-D3(BJ)/QZ4P. **S12**

Table S1. Activation strain analysis (in kcal mol^{-1}), bond lengths (in Å), change in the Y–H stretching frequency (in cm^{-1}), and change in the Voronoi deformation density charges on the NH_3 fragment for the $Me_mYH\cdots NH_3$ and $Me_mYH\cdots HNH_2$ complexes (Y = C, Si, Ge, N, P, As, O, S, Se; m = 3, 2, 1) at their equilibrium geometries. Computed at ZORA-BLYP-D3(BJ)/TZ2P.

Complex	ΔE	ΔE_{strain}	ΔE_{int}	$r_{H\cdots N}$	Δr_{Y-H}	Δr_{H-N}	$\Delta \upsilon_{Y-H}$	ΔQ_{NH3}
**$Me_3CH\cdots NH_3$**	–1.0	0.0	–1.0	2.689	–0.003	-	23	9
**$Me_2NH\cdots NH_3$**	–3.7	0.1	–3.8	2.204	0.005	-	–77	21
**$MeOH\cdots NH_3$**	–7.2	0.2	–7.5	1.932	0.016	-	–316	41
**$MeSH\cdots NH_3$**	–3.3	0.1	–3.4	2.230	0.010	-	–125	28
**$MeSeH\cdots NH_3$**	–2.9	0.1	–3.0	2.212	0.012	-	–134	34
**$Me_3CH\cdots HNH_2$**	–1.4	0.0	–1.4	2.187	0.004	0.000	–22	0
**$Me_3SiH\cdots HNH_2$**	–2.4	0.1	–2.5	2.202	0.007	0.000	–29	0
**$Me_3GeH\cdots HNH_2$**	–2.4	0.1	–2.5	2.214	0.001	–0.001	–38	0
**$Me_2PH\cdots HNH_2$**	–2.3	0.1	–2.4	2.198	0.003	0.000	–25	0
**$Me_2AsH\cdots HNH_2$**	–2.6	0.0	–2.6	2.098	0.008	0.001	–42	0

Table S2. Energy decomposition analysis (in kcal mol^{-1}) and populations (in electrons) in the empty σ^*_{Y-H} orbital for the $Me_mYH\cdots NH_3$ and $Me_mYH\cdots HNH_2$ complexes (Y = C, Si, Ge, N, P, As, O, S, Se; m = 3, 2, 1). Computed at ZORA-BLYP-D3(BJ)/TZ2P.

Complex	ΔE_{int}	ΔV_{elstat}	ΔE_{Pauli}	ΔE_{oi}	ΔE_{disp}	Pop. σ^*_{Y-H}
**$Me_3CH\cdots NH_3$**	–1.0	–1.3	2.1	–0.9	–1.0	0.01
**$Me_2NH\cdots NH_3$**	–3.8	–6.2	6.9	–3.1	–1.4	0.01
**$MeOH\cdots NH_3$**	–7.5	–12.1	12.8	–6.8	–1.3	0.04
**$MeSH\cdots NH_3$**	–3.4	–5.7	7.6	–3.9	–1.3	0.03
**$MeSeH\cdots NH_3$**	–3.0	–5.6	8.8	–4.7	–1.5	0.03
**$Me_3CH\cdots HNH_2$**	–1.4	–1.1	2.8	–1.1	–2.0	0.00
**$Me_3SiH\cdots HNH_2$**	–2.5	–3.0	4.6	–1.6	–2.5	0.00
**$Me_3GeH\cdots HNH_2$**	–2.5	–3.0	4.7	–1.7	–2.6	0.00
**$Me_2PH\cdots HNH_2$**	–2.4	–2.4	4.0	–1.7	–2.4	0.00
**$Me_2AsH\cdots HNH_2$**	–2.6	–2.9	4.9	–2.1	–2.6	0.00

Table S3. Activation strain analysis (in kcal mol^{-1}), bond lengths (in Å), change in the Y–H stretching frequency (in cm^{-1}), and change in the Voronoi deformation density charges on the ICN fragment for the $Me_mYH\cdots NCl$ and $Me_mYH\cdots ICN$ complexes (Y = C, Si, Ge, N, P, As, O, S, Se; m = 3, 2, 1) at their equilibrium geometries. Computed at ZORA-BLYP-D3(BJ)/TZ2P.

Complex	ΔE	ΔE_{strain}	ΔE_{int}	$r_{H\cdots I/N}$	Δr_{Y-H}	Δr_{I-C}	$\Delta \upsilon_{Y-H}$	ΔQ_{ICN}
**$Me_3CH\cdots NCl$**	–2.5	0.0	–2.5	2.793	–0.003	0.000	26	0
**$Me_2NH\cdots NCl$**	–2.6	0.0	–2.6	2.303	0.000	–0.001	9	8
**$MeOH\cdots NCl$**	–4.6	0.0	–4.6	2.058	0.004	–0.002	–86	20
**$MeSH\cdots NCl$**	–2.1	0.0	–2.1	2.444	0.000	0.000	1	7
**$MeSeH\cdots NCl$**	–2.0	0.1	–2.1	2.674	–0.003	0.000	15	2
**$Me_3CH\cdots ICN$**	–3.1	0.1	–3.2	2.871	0.003	0.004	–47	–11
**$Me_3SiH\cdots ICN$**	–5.4	0.3	–5.7	2.592	0.014	0.022	–87	–35
**$Me_3GeH\cdots ICN$**	–5.4	0.4	–5.8	2.557	0.019	0.026	–98	–39
**$Me_2PH\cdots ICN$**	–4.7	0.2	–4.9	2.629	0.010	0.019	–74	–33
**$Me_2AsH\cdots ICN$**	–5.4	0.2	–5.6	2.588	0.015	0.022	–85	–38

Table S4. Energy decomposition analysis (in kcal mol^{-1}) and populations (in electrons) in the occupied σ_{Y-H} and in the empty σ^*_{Y-H} and σ^*_{I-C} orbitals for the $Me_mYH\cdots NCl$ and $Me_mYH\cdots ICN$ complexes (Y = C, Si, Ge, N, P, As, O, S, Se; m = 3, 2, 1) at their equilibrium geometries. Computed at ZORA-BLYP-D3(BJ)/TZ2P.

Complex	ΔE_{int}	ΔV_{elstat}	ΔE_{Pauli}	ΔE_{oi}	ΔE_{disp}	Pop. σ_{Y-H}	Pop. σ^*_{Y-H}	Pop. σ^*_{I-C}
**$Me_3CH\cdots NCl$**	–2.5	–1.6	4.4	–1.3	–4.1	2.00	0.00	0.00
**$Me_2NH\cdots NCl$**	–2.6	–3.3	3.5	–1.6	–1.2	2.00	0.01	0.00
**$MeOH\cdots NCl$**	–4.6	–6.2	6.2	–3.4	–1.2	2.00	0.02	0.00
**$MeSH\cdots NCl$**	–2.1	–2.4	2.8	–1.3	–1.2	2.00	0.01	0.00
**$MeSeH\cdots NCl$**	–2.1	–1.9	2.7	–1.0	–1.9	2.00	0.00	0.00
**$Me_3CH\cdots ICN$**	–3.2	–1.6	4.9	–2.5	–4.0	1.99	0.00	0.02
**$Me_3SiH\cdots ICN$**	–5.7	–6.8	11.6	–6.3	–4.2	1.94	0.00	0.08
**$Me_3GeH\cdots ICN$**	–5.8	–7.4	12.9	–7.3	–4.1	1.93	0.01	0.09
**$Me_2PH\cdots ICN$**	–4.9	–4.2	9.9	–5.8	–4.8	1.97	0.01	0.07
**$Me_2AsH\cdots ICN$**	–5.6	–5.4	11.5	–6.8	–4.9	1.97	0.01	0.08

Table S5. HOMO–LUMO overlap, orbital energies (in eV), and magnitude of donor–acceptor interactions associated with the hydrogen and halogen bonds in the $Me_mYH\cdots NH_3$, $Me_mYH\cdots NCI$, and $Me_mYH\cdots ICN$ complexes (Y = C, Si, Ge, N, P, As, O, S, Se; m = 3, 2, 1). Computed at ZORA-BLYP-D3(BJ)/TZ2P.

	$\sigma^*_{Y-H} \leftarrow LP_{N/I}$					$\sigma_{Y-H} \rightarrow \sigma^*_{I-C}$				
**Complex**	S	$\varepsilon(\sigma^*_{Y-H})$	$\varepsilon(LP_{N/I})$	$\Delta\varepsilon$	$10^3 x S^2/\Delta\varepsilon$	S	$\varepsilon(\sigma_{Y-H})$	$\varepsilon(\sigma^*_{I-C})$	$\Delta\varepsilon$	$10^3 x S^2/\Delta\varepsilon$
**$Me_3CH\cdots NH_3$**	0.19	0.7	–6.0	6.7	5.4	-	-	-	-	-
**$Me_2NH\cdots NH_3$**	0.20	–0.2	–6.0	5.9	6.8	-	-	-	-	-
**$MeOH\cdots NH_3$**	0.29	–0.5	–6.0	5.6	15.4	-	-	-	-	-
**$MeSH\cdots NH_3$**	0.18	–0.8	–6.0	5.2	6.2	-	-	-	-	-
**$MeSeH\cdots NH_3$**	0.16	–1.1	–6.0	4.9	5.5	-	-	-	-	-
**$Me_3CH\cdots NCl$**	0.13	0.7	–8.9	9.6	1.6	-	-	-	-	-
**$Me_2NH\cdots NCl$**	0.22	–0.1	–8.9	8.8	5.4	-	-	-	-	-
**$MeOH\cdots NCl$**	0.33	–0.4	–8.9	8.5	12.9	-	-	-	-	-
**$MeSH\cdots NCl$**	0.18	–0.8	–8.9	8.2	4.1	-	-	-	-	-
**$Me_3CH\cdots ICN$**	0.14	0.7	–12.6	13.3	1.5	0.08	–7.5	–3.0	4.5	1.3
**$Me_3SiH\cdots ICN$**	0.15	0.7	–12.6	13.3	1.7	0.19	–7.1	–3.1	4.0	9.4
**$Me_3GeH\cdots ICN$**	0.19	0.4	–12.6	13.0	2.8	0.20	–7.0	–3.2	3.8	10.8
**$Me_2PH\cdots ICN$**	0.18	–0.4	–12.6	12.2	2.7	0.12	–8.1	–3.1	5.0	3.0
**$Me_2AsH\cdots ICN$**	0.18	–0.5	–12.6	12.1	2.7	0.15	–7.7	–3.1	4.6	4.7

Table S6. Activation strain and energy decomposition analyses (in kcal mol^{-1}), change in the Voronoi deformation density charge on the Me_3SiH fragments (in milli-electrons), and populations (in electrons) in the occupied σ_{Y-H} orbitals upon complexation with different Lewis acids. Computed at ZORA-BLYP-D3(BJ)/TZ2P.

**Complex**[a]	ΔE	ΔE_{strain}	ΔE_{int}	ΔV_{elstat}	ΔE_{Pauli}	ΔE_{oi}	ΔE_{disp}	ΔQ_{Me3SiH}	**Pop.** σ_{Y-H}
**$Me_3SiH\cdots ICF_3$**	–4.0	0.0	–4.0	–3.8	7.1	–3.0	–4.2	18	1.97
**$Me_3SiH\cdots BrCN$**	–4.0	0.0	–4.0	–3.7	6.4	–3.2	–3.5	17	1.98
**$Me_3SiH\cdots S(CN)_2$**	–5.2	0.1	–5.3	–5.2	9.1	–3.7	–5.6	18	1.97
**$Me_3SiH\cdots P(CN)_3$**	–7.2	0.2	–7.4	–8.1	14.7	–6.3	–7.7	39	1.95
**$Me_3SiH\cdots K^+$**	–12.5	0.7	–13.2	–7.3	3.6	–7.1	–2.4	22	1.94

[a] Geometries taken from Hobza *et. al.*[1]

(1) Civiš, S.; Lamanec, M.; Špirko, V.; Kubišta, J.; Špet'ko, M.; Hobza, P. Hydrogen Bonding with Hydridic Hydrogen–Experimental Low-Temperature IR and Computational Study: Is a Revised Definition of Hydrogen Bonding Appropriate? *J. Am. Chem. Soc.* **2023**, *145*, 8550-8559.

Table S7. Activation strain analysis (in kcal mol^{-1}), bond lengths (in Å), Voronoi deformation density charge on the H atom of the halogen-bond acceptor fragments in their equilibrium geometries (in milli-electrons), change in the Voronoi deformation density charges on the ICN fragment, and change in the Y–H stretching frequency (in cm^{-1}) for halogen-bonded systems with palladium hydride, boron hydride, beryllium hydride, and lithium hydride. Computed at ZORA-BLYP-D3(BJ)/TZ2P.

Complex	ΔE	ΔE_{strain}	ΔE_{int}	$r_{H\cdots I}$	Δr_{Y-H}	Q_H	ΔQ_{ICN}	$\Delta \upsilon_{Y-H}$
**$(PH_3)_2$MePdH•••ICN**	–10.3	1.2	–11.5	2.322	0.019	–222	–83	–31
**Me_2BH•••ICN**	–4.2	0.3	–4.4	2.593	0.012	–87	–29	–96
**MeBeH•••ICN**	–4.7	0.2	–4.8	2.593	0.007	–211	–33	–42
**LiH•••ICN**	–24.5	12.3	–36.7	2.006	0.137	–452	–152	–242

Table S8. Energy decomposition analysis (in kcal mol^{-1}) and populations (in electrons) in the occupied σ_{Y-H} orbital for halogen-bonded systems with palladium hydride, boron hydride, beryllium hydride, and lithium hydride. Computed at ZORA-BLYP-D3(BJ)/TZ2P.

Complex	ΔE_{int}	ΔV_{elstat}	ΔE_{Pauli}	ΔE_{oi}	ΔE_{disp}	Pop. σ_{Y-H}
**$(PH_3)_2$MePdH•••ICN**	–11.5	–16.7	26.6	–15.9	–5.5	1.90
**Me_2BH•••ICN**	–4.4	–6.0	10.3	–5.6	–3.1	1.95
**MeBeH•••ICN**	–4.9	–8.7	12.5	–6.6	–2.1	1.96
**LiH•••ICN**	–36.7	–107.3	150.6	–77.6	–2.4	1.43

Table S9. Cartesian coordinates (in Å), ADF total electronic energies (in kcal mol^{-1}), the Y–H stretching frequency (in cm^{-1}), and the number of imaginary vibrational frequencies for the Me_mYH, NH_3, and ICN fragments. Computed at ZORA-BLYP-D3(BJ)/QZ4P.

Me_3CH
***E* = -1633.42**
υ_{Y-H} = 2914
N_{imag} = 0

C	-0.73294100	1.26949100	0.06527800
C	0.00000000	0.00000000	-0.40668700
C	-0.73294100	-1.26949100	0.06527800
C	1.46588200	-0.00000000	0.06527800
H	-0.00000000	-0.00000000	-1.50823200
H	-1.76922600	1.28916100	-0.29506800
H	-0.75936800	1.31526400	1.16316400
H	-0.23183300	2.17677600	-0.29506800
H	-1.76922600	-1.28916100	-0.29506800
H	-0.23183300	-2.17677600	-0.29506800
H	-0.75936800	-1.31526400	1.16316400
H	2.00106000	0.88761400	-0.29506800
H	1.51873600	-0.00000000	1.16316400
H	2.00106000	-0.88761400	-0.29506800

Me_3SiH
***E* = -1551.59**
υ_{Y-H} = 2128
N_{imag} = 0

C	-0.89486500	1.54995100	0.11057300
Si	0.00000000	0.00000000	-0.49612500
C	-0.89486500	-1.54995100	0.11057300
C	1.78973000	-0.00000000	0.11057300
H	-0.00000000	-0.00000000	-1.99214100
H	-1.93279900	1.57452600	-0.24452800
H	-0.91497800	1.58478800	1.20790500
H	-0.39718000	2.46111600	-0.24452800
H	-1.93279900	-1.57452600	-0.24452800
H	-0.39718000	-2.46111600	-0.24452800
H	-0.91497800	-1.58478800	1.20790500
H	2.32997900	0.88659000	-0.24452800
H	1.82995600	-0.00000000	1.20790500
H	2.32997900	-0.88659000	-0.24452800

Me_3GeH
***E* = -1507.96**
υ_{Y-H} = 2008
N_{imag} = 0

C	-0.93666700	1.62235400	0.12189300
Ge	0.00000000	0.00000000	-0.53052900
C	-0.93666700	-1.62235400	0.12189300
C	1.87333300	-0.00000000	0.12189300
H	-0.00000000	-0.00000000	-2.08106300
H	-1.97206800	1.63582300	-0.23444500
H	-0.94239100	1.63226900	1.21752700
H	-0.43063100	2.52577300	-0.23444500
H	-1.97206800	-1.63582300	-0.23444500
H	-0.43063100	-2.52577300	-0.23444500
H	-0.94239100	-1.63226900	1.21752700
H	2.40269900	0.88994900	-0.23444500
H	1.88478200	-0.00000000	1.21752700
H	2.40269900	-0.88994900	-0.23444500

Me_2NH
***E* = -1152.33**
υ_{Y-H} = 3406
N_{imag} = 0

H	0.63676800	-1.02433200	-1.29039900
N	0.37498900	0.61484500	0.00000000
H	-1.04044000	-0.42864700	1.26919900
H	-0.09494500	1.52024100	0.00000000
C	0.02239300	-0.11728900	1.22288700
H	0.63676800	-1.02433200	1.29039900
H	0.24125700	0.50272500	2.09908200
C	0.02239300	-0.11728900	-1.22288700
H	-1.04044000	-0.42864700	-1.26919900
H	0.24125700	0.50272500	-2.09908200

Me_2PH
***E* = -1085.09**
υ_{Y-H} = 2281
N_{imag} = 0

H	0.69086400	-1.05977800	-1.50744600
P	0.64862900	0.87733300	0.00000000
H	-0.99081900	-0.49619500	1.31638500
H	-0.44759800	1.79524900	0.00000000
C	0.04997700	-0.17391500	1.43267900
H	0.69086400	-1.05977800	1.50744600
H	0.14946200	0.39359700	2.36431300
C	0.04997700	-0.17391500	-1.43267900
H	-0.99081900	-0.49619500	-1.31638500
H	0.14946200	0.39359700	-2.36431300

Me_2AsH

***E* = -1059.55**

υ_{Y-H} = 2049

N_{imag} = 0

H	0.70796700	-1.07251900	-1.56105800
As	0.71361700	0.97241700	0.00000000
H	-0.97641200	-0.51628900	1.33324200
H	-0.52815500	1.88171300	0.00000000
C	0.05471100	-0.19644100	1.50337500
H	0.70796700	-1.07251900	1.56105800
H	0.12100300	0.35818400	2.44386200
C	0.05471100	-0.19644100	-1.50337500
H	-0.97641200	-0.51628900	-1.33324200
H	0.12100300	0.35818400	-2.44386200

MeOH

***E* = -672.23**

υ_{Y-H} = 3678

N_{imag} = 0

H	-0.58603900	-0.58812800	0.89674300
O	0.51356300	0.98239900	0.00000000
H	-0.58603900	-0.58812800	-0.89674300
H	-0.24698100	1.58754800	0.00000000
C	0.01383200	-0.37021200	0.00000000
H	0.89166400	-1.02347900	0.00000000

MeSH

***E* = -609.47**

υ_{Y-H} = 2573

N_{imag} = 0

H	-0.58899200	-0.67557400	0.89834700
S	0.74134200	1.18863800	0.00000000
H	-0.58899200	-0.67557400	-0.89834700
H	-0.43074600	1.86683600	0.00000000
C	0.00957900	-0.50737700	0.00000000
H	0.85781000	-1.19694900	0.00000000

MeSeH

***E* = -589.20**

υ_{Y-H} = 2294

N_{imag} = 0

H	-0.59231800	-0.70663400	0.90051600
Se	0.81496800	1.26313200	0.00000000
H	-0.59231800	-0.70663400	-0.90051600
H	-0.48730200	1.97183400	0.00000000
C	0.00587000	-0.56513100	0.00000000
H	0.85109900	-1.25656500	0.00000000

$(PH_3)_2MePdH$

***E* = -1282.72**

υ_{Y-H} = 1813

N_{imag} = 0

H	0.54713500	-1.40265800	0.00086300
Pd	-0.23449200	-0.00723500	-0.00137000
C	-1.90975900	-1.31572100	-0.00026000
H	-2.49945500	-1.08727900	-0.89625500
H	-1.63618100	-2.37005500	0.00020900
H	-2.49741800	-1.08580000	0.89670100
P	-1.65782400	1.87734000	-0.00016300
H	-1.62374900	2.83098300	-1.05567600
H	-3.06587100	1.68522800	0.00019200
H	-1.62317100	2.83034500	1.05592300
P	1.87473000	0.99032800	-0.00096800
H	2.15318000	2.38921900	-0.00004900
H	2.76048100	0.66270600	1.05915100
H	2.76418800	0.66363000	-1.05841700

Me_2BeH

***E* = -1116.55**

υ_{Y-H} = 2502

N_{imag} = 0

H	-0.00000000	0.00000000	-1.78002600
B	0.00000000	-0.00000000	-0.57675100
C	0.05198700	1.37759200	0.16611600
C	-0.05198700	-1.37759200	0.16611600
H	0.31551000	1.32005300	1.23012800
H	-0.97282800	1.79297900	0.11491900
H	0.68499600	2.12364100	-0.33277400
H	0.97282800	-1.79297900	0.11491900
H	-0.68499600	-2.12364100	-0.33277400
H	-0.31551000	-1.32005300	1.23012800

MeBeH

***E* = -578.36**

υ_{Y-H} = 2120

N_{imag} = 0

H	-0.00000000	-0.00000000	2.43293100
Be	0.00000000	0.00000000	1.10142300
C	0.00000000	0.00000000	-0.57029100
H	-0.50957000	0.88260200	-0.98802100
H	-0.50957000	-0.88260200	-0.98802100
H	1.01914100	0.00000000	-0.98802100

LiH

***E* = -85.26**

υ_{Y-H} = 1387

N_{imag} = 0

H	0.00000000	0.00000000	-0.79865000
Li	0.00000000	0.00000000	0.79865000

NH_3
***E* = -434.94**
N_{imag} = 0

H	-0.47340500	0.81996200	-0.09603800
N	0.00000000	0.00000000	0.28811400
H	0.94681000	0.00000000	-0.09603800
H	-0.47340500	-0.81996200	-0.09603800

ICN
***E* = -374.62**
N_{imag} = 0

I	0.00000000	-0.00000000	1.73328500
C	-0.00000000	0.00000000	-0.28356400
N	0.00000000	-0.00000000	-1.44972100

Table S10. Cartesian coordinates (in Å), ADF total electronic energies (in kcal mol^{-1}), the Y–H stretching frequency (in cm^{-1}), and the number of imaginary vibrational frequencies for the $Me_mYH{\cdots}NH_3$ and $Me_mYH{\cdots}HNH_2$ complexes. Computed at ZORA-BLYP-D3(BJ)/QZ4P.

$Me_3CH{\cdots}NH_3$
***E* = -2069.35**
υ_{Y-H} = 2937
N_{imag} = 0

H	0.51411000	0.00265500	0.00000000
C	-0.58442500	-0.02766900	0.00000000
C	-1.01495800	-1.50611800	0.00000000
C	-1.08124000	0.69063300	1.26873700
C	-1.08124000	0.69063300	-1.26873700
H	-0.63632500	-2.02955500	-0.88698100
H	-2.11079100	-1.59527700	0.00000000
H	-0.63632500	-2.02955500	0.88698100
H	-0.70780800	0.19866500	2.17580800
H	-0.74752400	1.73597400	1.29035200
H	-0.74752400	1.73597400	-1.29035200
H	-0.70780800	0.19866500	-2.17580800
H	-2.17965800	0.68931200	1.31473500
H	-2.17965800	0.68931200	-1.31473500
H	3.30491000	1.14977000	0.00000000
N	3.20022100	0.13334400	0.00000000
H	3.69923000	-0.21515700	0.82054000
H	3.69923000	-0.21515700	-0.82054000

$Me_2NH{\cdots}NH_3$
***E* = -1590.99**
υ_{Y-H} = 3329
N_{imag} = 0

H	-2.31379300	0.09211400	1.28798100
N	-0.70584000	0.54007200	0.00000000
H	-1.07162600	-1.18136000	-1.26441700
H	0.31616100	0.46202800	0.00000000
C	-1.23150300	-0.08326800	-1.21737500
H	-2.31379300	0.09211400	-1.28798100
H	-0.75975900	0.36997500	-2.09688300
C	-1.23150300	-0.08326800	1.21737500
H	-1.07162600	-1.18136000	1.26441700
H	-0.75975900	0.36997500	2.09688300
H	2.84479700	-0.32931100	-0.82069200
H	2.95226800	1.08906200	0.00000000
N	2.50118000	0.17254000	0.00000000
H	2.84479700	-0.32931100	0.82069200

$MeOH{\cdots}NH_3$
***E* = -1114.41**
υ_{Y-H} = 3362
N_{imag} = 0

H	-1.28861600	-1.08849800	-0.89460100
O	-0.82690200	0.76968000	0.00000000
H	-1.28861600	-1.08849800	0.89460100
H	0.14361200	0.58692100	0.00000000
C	-1.51679200	-0.48433700	0.00000000
H	-2.59076100	-0.26788500	0.00000000
N	2.06020700	0.34556900	0.00000000
H	2.41535000	-0.14373300	0.82291900
H	2.41535000	-0.14373300	-0.82291900
H	2.47838500	1.27749900	0.00000000

$MeSH{\cdots}NH_3$
***E* = -1047.73**
υ_{Y-H} = 2448
N_{imag} = 0

H	-1.44940100	-1.17881100	-0.89702000
S	-1.16747500	1.09666400	0.00000000
H	-1.44940100	-1.17881100	0.89702000
H	0.16322300	0.79776900	0.00000000
C	-1.77781600	-0.64687800	0.00000000
H	-2.86989500	-0.59050400	0.00000000
N	2.35609500	0.39422600	0.00000000
H	2.68354500	-0.11518800	0.82245400
H	2.68354500	-0.11518800	-0.82245400
H	2.82880100	1.29970500	0.00000000

$MeSeH{\cdots}NH_3$
***E* = -1027.06**
υ_{Y-H} = 2160
N_{imag} = 0

H	-1.34579500	-1.17790500	-0.89932900
Se	-1.28632600	1.24365500	0.00000000
H	-1.34579500	-1.17790500	0.89932900
H	0.18866300	1.00161000	0.00000000
C	-1.74241400	-0.70405900	0.00000000
H	-2.83329500	-0.76106000	0.00000000
N	2.32135400	0.41447100	0.00000000
H	2.54653700	-0.14687500	0.82284000
H	2.54653700	-0.14687500	-0.82284000
H	2.95175500	1.21793000	0.00000000

$Me_3CH \cdots HNH_2$
***E* = -2069.74**
υ_{Y-H} = 2892
N_{imag} = 0

C	-0.12429500	0.53084500	0.00000000
C	0.67360600	0.17759500	-1.26952600
C	-0.54449000	2.01236500	0.00000000
H	0.95262500	-0.88235400	-1.28077000
H	0.09390000	0.39187000	-2.17641800
H	-1.14049200	2.25811900	-0.88779700
H	-1.14049200	2.25811900	0.88779700
H	1.59803400	0.76962300	-1.31653100
H	-1.04842900	-0.07154200	0.00000000
C	0.67360600	0.17759500	1.26952600
H	0.09390000	0.39187000	2.17641800
H	1.59803400	0.76962300	1.31653100
H	0.95262500	-0.88235400	1.28077000
H	0.34078100	2.66302700	0.00000000
H	-0.82496100	-3.67063900	-0.81966600
N	-0.45162700	-3.18805400	0.00000000
H	-0.82496100	-3.67063900	0.81966600
H	-0.85596000	-2.25021100	0.00000000

$Me_3SiH \cdots HNH_2$
***E* = -1988.97**
υ_{Y-H} = 2099
N_{imag} = 0

Si	-0.22684000	0.52314900	0.00000000
C	0.73704600	0.04064500	-1.54912600
C	-0.60917100	2.37381300	0.00000000
H	0.94606500	-1.03527300	-1.54079300
H	0.18125600	0.28184700	-2.46397700
H	-1.18716900	2.66168400	-0.88693300
H	-1.18716900	2.66168400	0.88693300
H	1.69611400	0.57432700	-1.59057000
H	-1.53141600	-0.21774400	0.00000000
C	0.73704600	0.04064500	1.54912600
H	0.18125600	0.28184700	2.46397700
H	1.69611400	0.57432700	1.59057000
H	0.94606500	-1.03527300	1.54079300
H	0.31965400	2.95901000	0.00000000
H	-0.58413700	-3.70143700	-0.81949300
N	-0.40252500	-3.11895900	0.00000000
H	-0.58413700	-3.70143700	0.81949300
H	-1.10664800	-2.37799700	0.00000000

$Me_3GeH \cdots HNH_2$
***E* = -1945.35**
υ_{Y-H} = 1970
N_{imag} = 0

Ge	-0.25409300	0.51830400	0.00000000
C	0.76035800	0.00632100	-1.62227400
C	-0.63270700	2.46686500	0.00000000
H	0.96361000	-1.06816600	-1.59209800
H	0.19104100	0.24434700	-2.52675700
H	-1.20673000	2.74457700	-0.89012100
H	-1.20673000	2.74457700	0.89012100
H	1.71127000	0.54949700	-1.65188600
H	-1.61480900	-0.23952100	0.00000000
C	0.76035800	0.00632100	1.62227400
H	0.19104100	0.24434700	2.52675700
H	1.71127000	0.54949700	1.65188600
H	0.96361000	-1.06816600	1.59209800
H	0.30843200	3.02756900	0.00000000
H	-0.56685900	-3.71079800	-0.81948400
N	-0.38929400	-3.12673900	0.00000000
H	-0.56685900	-3.71079800	0.81948400
H	-1.10150700	-2.39317700	0.00000000

$Me_2PH \cdots HNH_2$
***E* = -1522.36**
υ_{Y-H} = 2256
N_{imag} = 0

H	1.66467300	-0.23766100	0.00000000
P	1.31721000	1.15267300	0.00000000
H	-0.38193100	2.05302000	1.52017700
H	-0.65024100	0.30094200	-1.29672200
C	0.10970100	1.07727100	-1.43210300
H	-0.38193100	2.05302000	-1.52017700
H	0.66145400	0.89553700	-2.36132900
C	0.10970100	1.07727100	1.43210300
H	-0.65024100	0.30094200	1.29672200
H	0.66145400	0.89553700	2.36132900
N	-0.80327000	-2.17449300	0.00000000
H	-0.91027200	-2.77538300	-0.81946700
H	0.16396700	-1.84329400	0.00000000
H	-0.91027200	-2.77538300	0.81946700

$Me_2AsH \cdots HNH_2$
***E* = -1497.07**
υ_{Y-H} = 2007
N_{imag} = 0

H	1.70741700	-0.36242000	0.00000000
As	1.42039500	1.15743500	0.00000000
H	-0.39738500	2.05864800	1.56903500
H	-0.66681500	0.30612900	-1.30854000
C	0.08191000	1.07733800	-1.50163200
H	-0.39738500	2.05864800	-1.56903500
H	0.60891800	0.87376000	-2.43824100
C	0.08191000	1.07733800	1.50163200
H	-0.66681500	0.30612900	1.30854000
H	0.60891800	0.87376000	2.43824100
N	-0.78601900	-2.14195900	0.00000000
H	-0.88592700	-2.74380700	-0.81962900
H	0.17680500	-1.79719300	0.00000000
H	-0.88592700	-2.74380700	0.81962900

Table S11. Cartesian coordinates (in Å), ADF total electronic energies (in kcal mol^{-1}), the Y–H stretching frequency (in cm^{-1}), and the number of imaginary vibrational frequencies for Me$_m$YH•••NCI and Me$_m$YH•••ICN complexes. Computed at ZORA-BLYP-D3(BJ)/QZ4P.

Me$_3$CH•••NCI
***E* = -2010.56**
υ_{Y-H} = 2940
N_{imag} = 0

H	1.08588400	-0.40468800	0.00000000
C	0.01104900	-0.63489500	0.00000000
C	-0.14932200	-2.16687800	0.00000000
C	-0.60333400	-0.01703500	1.26963400
C	-0.60333400	-0.01703500	-1.26963400
H	0.31531400	-2.61452600	-0.88724900
H	-1.21218100	-2.44664100	0.00000000
H	0.31531400	-2.61452600	0.88724900
H	-0.14484100	-0.43271700	2.17521800
H	-0.46452200	1.07046000	1.29230200
H	-0.46452200	1.07046000	-1.29230200
H	-0.14484100	-0.43271700	-2.17521800
H	-1.68309500	-0.21674300	1.31658800
H	-1.68309500	-0.21674300	-1.31658800
N	2.77647600	1.81839800	0.00000000
C	1.93448500	2.62481600	0.00000000
I	0.46571800	4.00429800	0.00000000

Me$_2$NH•••NCI
***E* = -1529.51**
υ_{Y-H} = 3415
N_{imag} = 0

H	0.75879600	0.09755200	0.00000000
H	-1.19624800	1.02870000	-1.26196400
N	0.56398000	1.09905700	0.00000000
H	-0.25883800	2.54202400	1.29532000
C	-0.17019100	1.44968900	1.21968900
H	-1.19624800	1.02870000	1.26196400
H	0.38360000	1.09511900	2.09620300
C	-0.17019100	1.44968900	-1.21968900
H	-0.25883800	2.54202400	-1.29532000
H	0.38360000	1.09511900	-2.09620300
N	1.18354400	-2.16622600	0.00000000
C	1.40353900	-3.31053500	0.00000000
I	1.78789400	-5.28872400	0.00000000

MeOH•••NCI
***E* = -1051.41**
υ_{Y-H} = 3592
N_{imag} = 0

H	0.31422100	0.80346000	0.00000000
H	1.54810300	-1.00357300	0.89494200
O	1.28658400	0.89281600	0.00000000
H	1.54810300	-1.00357300	-0.89494200
C	1.84083900	-0.43083200	0.00000000
H	2.93065600	-0.32287900	0.00000000
N	-1.71429200	0.45777900	0.00000000
C	-2.87825300	0.42452900	0.00000000
I	-4.89073900	0.36831400	0.00000000

MeSH•••NCI
***E* = -986.20**
υ_{Y-H} = 2574
N_{imag} = 0

H	0.31297000	0.90734200	0.00000000
H	1.62113700	-1.25811200	0.89672900
S	1.66115900	1.03032400	0.00000000
H	1.62113700	-1.25811200	-0.89672900
C	2.02354300	-0.78026400	0.00000000
H	3.11280000	-0.87439600	0.00000000
N	-2.06231900	0.33145100	0.00000000
C	-3.17965100	0.66368500	0.00000000
I	-5.11077500	1.23808100	0.00000000

MeSeH•••NCI
***E* = -965.84**
υ_{Y-H} = 2309
N_{imag} = 0

H	0.33543600	1.44019100	0.00000000
H	1.20179800	-1.06588800	0.89678300
Se	1.80634700	1.27230900	0.00000000
H	1.20179800	-1.06588800	-0.89678300
C	1.71736200	-0.72387900	0.00000000
H	2.75501100	-1.06469500	0.00000000
N	-1.72170900	-0.26880600	0.00000000
C	-2.75559100	0.27057700	0.00000000
I	-4.54045200	1.20607900	0.00000000

$Me_3CH\cdots ICN$
***E* = -2011.16**
υ_{Y-H} = 2867
N_{imag} = 0

H	0.87220100	-0.22342500	0.00000000
C	-0.12171100	-0.70582400	0.00000000
C	0.12577000	-2.22547700	0.00000000
C	-0.87034600	-0.26499100	1.27139600
C	-0.87034600	-0.26499100	-1.27139600
H	0.68880500	-2.53788800	-0.88779400
H	-0.82983500	-2.76731300	0.00000000
H	0.68880500	-2.53788800	0.88779400
H	-0.31274100	-0.53335400	2.17681000
H	-1.03995600	0.81889200	1.28649100
H	-1.03995600	0.81889200	-1.28649100
H	-0.31274100	-0.53335400	-2.17681000
H	-1.85232300	-0.75394000	1.32430300
H	-1.85232300	-0.75394000	-1.32430300
I	1.53294800	2.57009200	0.00000000
C	2.35848500	4.41514300	0.00000000
N	2.83526300	5.47936600	0.00000000

$Me_3SiH\cdots ICN$
***E* = -1931.60**
υ_{Y-H} = 2041
N_{imag} = 0

H	1.25125600	-0.01919100	0.00000000
Si	-0.08123800	-0.73055200	0.00000000
C	0.27858900	-2.58042900	0.00000000
C	-1.00517300	-0.20465300	1.55471900
C	-1.00517300	-0.20465300	-1.55471900
H	0.85210700	-2.87292600	-0.88757400
H	-0.65863400	-3.15157500	0.00000000
H	0.85210700	-2.87292600	0.88757400
H	-0.43429400	-0.44913200	2.45858300
H	-1.19558000	0.87512200	1.55664200
H	-1.19558000	0.87512200	-1.55664200
H	-0.43429400	-0.44913200	-2.45858300
H	-1.97369800	-0.71704400	1.61972900
H	-1.97369800	-0.71704400	-1.61972900
I	1.82176700	2.50897400	0.00000000
C	2.28863500	4.49364900	0.00000000
N	2.55308600	5.62934500	0.00000000

$Me_3GeH\cdots ICN$
***E* = -1887.96**
υ_{Y-H} = 1910
N_{imag} = 0

H	1.29968800	0.06569200	0.00000000
Ge	-0.09678500	-0.65115200	0.00000000
C	0.24731700	-2.59915600	0.00000000
C	-1.06110200	-0.08272500	1.63077300
C	-1.06110200	-0.08272500	-1.63077300
H	0.81498300	-2.88565700	-0.89075200
H	-0.70696200	-3.13756200	0.00000000
H	0.81498300	-2.88565700	0.89075200
H	-0.49138400	-0.35465500	2.52481100
H	-1.21103000	1.00162200	1.62197400
H	-1.21103000	1.00162200	-1.62197400
H	-0.49138400	-0.35465500	-2.52481100
H	-2.03921400	-0.57432500	1.67118300
H	-2.03921400	-0.57432500	-1.67118300
I	1.91811300	2.54677100	0.00000000
C	2.46343000	4.51590300	0.00000000
N	2.77359700	5.64029300	0.00000000

$Me_2PH\cdots ICN$
***E* = -1464.40**
υ_{Y-H} = 2207
N_{imag} = 0

H	1.27606800	0.68178900	0.00000000
H	-1.13160800	0.82255400	-1.31656800
P	0.66513400	1.98601000	0.00000000
H	-1.14583700	2.59197600	1.52474800
C	-0.50136200	1.71008900	1.43552300
H	-1.13160800	0.82255400	1.31656800
H	0.07756900	1.62114900	2.36094700
C	-0.50136200	1.71008900	-1.43552300
H	-1.14583700	2.59197600	-1.52474800
H	0.07756900	1.62114900	-2.36094700
I	0.46635400	-1.81905000	0.00000000
C	-0.12137600	-3.76809400	0.00000000
N	-0.45916900	-4.88431200	0.00000000

$Me_2AsH\cdots ICN$
***E* = -1439.58**
υ_{Y-H} = 1964
N_{imag} = 0

H	1.35826200	0.61369400	0.00000000
H	-1.13079600	0.82846500	-1.32705200
As	0.76476600	2.05015200	0.00000000
H	-1.16712900	2.59706700	1.59049900
C	-0.52250400	1.71754900	1.50718800
H	-1.13079600	0.82846500	1.32705200
H	0.04354600	1.59990000	2.43543800
C	-0.52250400	1.71754900	-1.50718800
H	-1.16712900	2.59706700	-1.59049900
H	0.04354600	1.59990000	-2.43543800
I	0.48918200	-1.82356900	0.00000000
C	-0.13700300	-3.76450800	0.00000000
N	-0.49690600	-4.87385200	0.00000000

$(PH_3)_2MePdH\cdots ICN$
***E* = -1667.66**
υ_{Y-H} = 1782
N_{imag} = 0

H	-1.55695400	2.77839000	-1.06106500
H	-3.02674800	1.65141600	0.00000000
P	-1.61972800	1.83557000	0.00000000
H	-1.55695400	2.77839000	1.06106500
H	2.06939500	2.42906800	0.00000000
H	2.76843700	0.72516100	1.06281200
I	2.38796400	-2.84372800	0.00000000
C	4.04693800	-4.09906100	0.00000000
N	4.98229500	-4.79619400	0.00000000
H	2.76843700	0.72516100	-1.06281200
C	-1.93880900	-1.29458900	0.00000000
Pd	-0.22924100	-0.04230700	0.00000000
H	0.50545800	-1.48438300	0.00000000
H	-2.51870200	-1.05476300	0.89802500
H	-1.67157600	-2.35009000	0.00000000
H	-2.51870200	-1.05476300	-0.89802500
P	1.87748300	1.01877200	0.00000000

$Me_2BH\cdots ICN$
***E* = -1495.36**
υ_{Y-H} = 2406
N_{imag} = 0

H	1.07100800	-0.16705900	0.00951100
B	0.39601500	0.84274300	0.00497000
C	-0.01798500	1.40599800	-1.38847400
C	0.02522900	1.44801900	1.39261800
H	-0.74354400	2.22758900	-1.35857500
H	-0.38186000	0.62004300	-2.06619100
H	0.89865300	1.78799900	-1.87483200
H	0.85991700	1.42374800	2.10738200
H	-0.74051100	0.78336100	1.83518000
H	-0.40135000	2.45759200	1.35708300
I	0.24690200	-2.62536900	-0.00092100
C	-0.41619400	-4.55110700	-0.01002400
N	-0.79628100	-5.65355800	-0.01518100

$MeBeH\cdots ICN$
***E* = -957.68**
υ_{Y-H} = 2078
N_{imag} = 0

H	0.95965261	1.57088125	0.00126855
Be	0.16008382	2.64346662	0.05296550
C	-0.82905842	3.98083515	0.11711307
H	-0.28658159	4.88858437	0.42361289
H	-1.65564265	3.85217604	0.83304462
H	-1.29038204	4.20473962	-0.85721341
I	0.33233255	-0.94505214	0.01862211
C	-0.15261747	-2.92924015	0.02841967
N	-0.43186758	-4.06141835	0.03326226

$LiH\cdots ICN$
***E* = -484.35**
υ_{Y-H} = 1145
N_{imag} = 0

H	0.78608100	-0.34631800	2.25782900
Li	-0.79410100	0.35125700	2.41316700
I	0.46210400	-0.20539000	0.28343500
C	-0.12020300	0.05186100	-1.92626100
N	-0.47364200	0.21122900	-3.02817000
